# Genomic evolution of antimicrobial resistance in *Escherichia coli*

**DOI:** 10.1038/s41598-021-93970-7

**Published:** 2021-07-23

**Authors:** Pimlapas Leekitcharoenphon, Markus Hans Kristofer Johansson, Patrick Munk, Burkhard Malorny, Magdalena Skarżyńska, Katharina Wadepohl, Gabriel Moyano, Ayla Hesp, Kees T. Veldman, Alex Bossers, EFFORT Consortium, Haitske Graveland, Alieda van Essen, Antonio Battisti, Andrea Caprioli, Thomas Blaha, Tine Hald, Hristo Daskalov, Helmut W. Saatkamp, Katharina D. C. Stärk, Roosmarijn E. C. Luiken, Liese Van Gompel, Rasmus Borup Hansen, Jeroen Dewulf, Ana Sofia Ribeiro Duarte, Magdalena Zając, Dariusz Wasyl, Pascal Sanders, Bruno Gonzalez-Zorn, Michael S. M. Brouwer, Jaap A. Wagenaar, Dick J. J. Heederik, Dik Mevius, Frank M. Aarestrup

**Affiliations:** 1 National Food Institute, Technical University of Denmark, Kemitorvet, Building 204, 2800 Kgs. Lyngby, Denmark; 2 Department Biological Safety, German Federal Institute for Risk Assessment, Berlin, Germany; 3 National Veterinary Research Institute, Puławy, Poland; 4 University of Veterinary Medicine Hannover, Bakum, Germany; 5 Department of Animal Health and Health Surveillance Center (VISAVET), Complutense University of Madrid, Madrid, Spain; 6 Wageningen Bioveterinary Research, Lelystad, The Netherlands; 7 Faculty of Veterinary Medicine, Institute for Risk Assessment Sciences, Utrecht University, Utrecht, The Netherlands; 8 Fougeres Laboratory, French Agency for Food, Environmental and Occupational Health and Safety, Fougères, France; 9 Department of Infectious Diseases and Immunology, Utrecht University, Utrecht, The Netherlands; 10 Department of General Diagnostics, Istituto Zooprofilattico Sperimentale del Lazio E Della Toscana, National Reference Laboratory for Antimicrobial Resistance, Rome, Italy; 11 National Diagnostic Research Veterinary Institute, Sofia, Bulgaria; 12 SAFOSO AG, Liebefeld, Switzerland; 13 Intomics A/S, Diplomvej 377, Kongens Lyngby, Denmark; 14 Department of Reproduction, Obstetrics and Herd Health, Ghent University, Merelbeke, Belgium

**Keywords:** Antimicrobial resistance, Molecular evolution

## Abstract

The emergence of antimicrobial resistance (AMR) is one of the biggest health threats globally. In addition, the use of antimicrobial drugs in humans and livestock is considered an important driver of antimicrobial resistance. The commensal microbiota, and especially the intestinal microbiota, has been shown to have an important role in the emergence of AMR. Mobile genetic elements (MGEs) also play a central role in facilitating the acquisition and spread of AMR genes. We isolated *Escherichia coli* (n = 627) from fecal samples in respectively 25 poultry, 28 swine, and 15 veal calf herds from 6 European countries to investigate the phylogeny of *E. coli* at country, animal host and farm levels. Furthermore, we examine the evolution of AMR in *E. coli* genomes including an association with virulence genes, plasmids and MGEs. We compared the abundance metrics retrieved from metagenomic sequencing and whole genome sequenced of *E. coli* isolates from the same fecal samples and farms. The *E. coli* isolates in this study indicated no clonality or clustering based on country of origin and genetic markers; AMR, and MGEs. Nonetheless, mobile genetic elements play a role in the acquisition of AMR and virulence genes. Additionally, an abundance of AMR was agreeable between metagenomic and whole genome sequencing analysis for several AMR classes in poultry fecal samples suggesting that metagenomics could be used as an indicator for surveillance of AMR in *E. coli* isolates and vice versa.

## Introduction

*Escherichia coli*, is an inhabitant of the gastrointestinal tract and feces of warm-blooded animals and reptiles^1,2^. *Escherichia coli* has the interesting characteristic of being both a widespread gut commensal bacteria in vertebrates and a versatile pathogen, resulting in killing more than 2 million humans per year through both intra- and extraintestinal diseases^3,4^. The genetic structure of commensal *E. coli* is shaped by multiple host and environmental factors, and genetic determinants involved in the virulence of the bacteria reflect adaptation to commensal habitats^5^. Selective pressure in the habitats of commensal strains may coincidentally promote the emergence of virulence factors and antimicrobial resistance (AMR), rendering commensal *E. coli* strain reservoirs of virulence and AMR determinants^5^. Emergence of antimicrobial resistance is one of the biggest global threat to public health^6^. In addition to the use of antimicrobials in humans, the use of antimicrobials in livestock is considered an important driver of AMR, subsequently compromising human health^7^.

Besides AMR in zoonotic pathogens, AMR in commensal bacteria is perturbing due to its ability to spread horizontally to pathogens^8^. The commensal microbiota, and especially the intestinal microbiota, has shown to play an important role in the emergence of antimicrobial resistance^9^. The extensive use of antimicrobials in both human and veterinary medicine, trigger the selection of antimicrobial resistance in the commensal microorganisms^5^. Antimicrobial resistance genes are frequently mobilized and disseminated through the bacterial population through the interplay of various mobile genetic elements (MGEs) of different types^10^.

In this study, we isolated *E. coli* from fecal samples in poultry flocks, swine, and veal calve herds from 6 European countries to investigate phylogenetic background and clustering of *E. coli* at country, animal source and farm levels. Furthermore, this study provided an insight into the evolution of AMR in *E. coli* genomes including an association with virulence genes, plasmid and mobile genetic elements (MGEs). In addition, the correlation of AMR abundance between metagenomics and WGS analyses was determined.

## Results

### *Escherichia coli* phylogeny and genetic diversity

**Figure 1:**
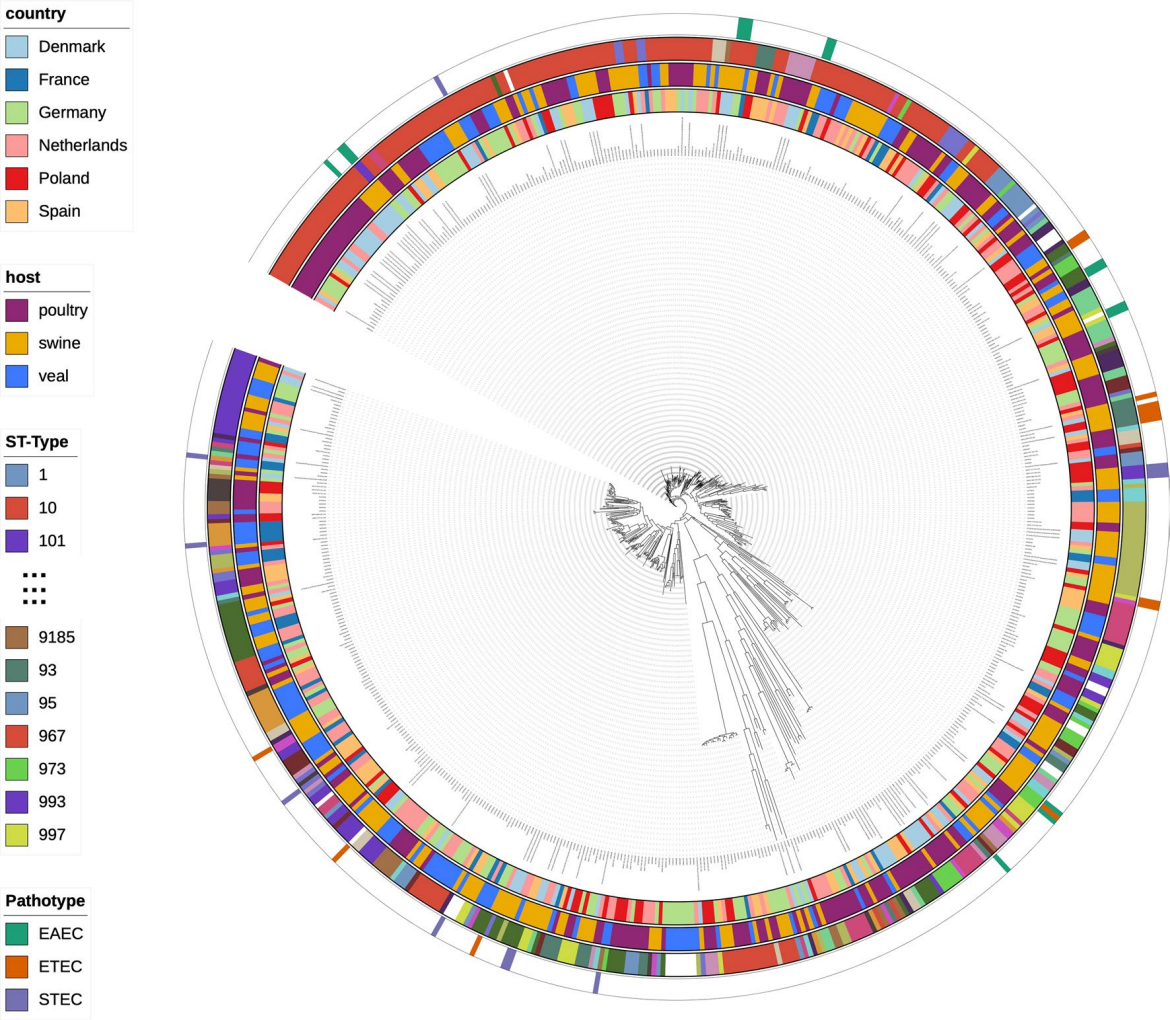
SNP tree of 627 *E. coli* isolates. The tree was constructed from 17,213 SNPs. The legends from inner circle to outer circle are country, host, ST type and pathotype. There are 182 different ST types with 24 isolates characterized as unknown ST type. Only a number of ST type can be showed in figure legend. *EAEC* Enteroaggregative *E. coli*, *ETEC* Enterotoxigenic *E. coli* and *STEC* Shiga toxin-producing *E. coli*.

The phylogenetic SNP tree exhibited diverse strains of *E. coli* with 17,213 total SNPs (Fig. 1). There was no clear clustering either by country of origin or animal host. In addition, within a small cluster from the same animal host, it contained strains from more than one country. Most of *E. coli* isolates were non-pathogenic strains. There were 39 isolates (6%) identified as EAEC/Enteroaggregative *E. coli* (n = 15, 2%), ETEC/ Enterotoxigenic *E. coli* (n = 13, 2%) and STEC/Shiga toxin-producing *E. coli* (n = 11, 2%). Furthermore, the SNP tree did not show any country and animal reservoir clusters of AMR genes nor chromosomal point mutation conferring to antimicrobial resistance (Figs. S2–S4). Looking at SNP tree in a country level (Figs. S5–S10), *E. coli* isolates within the same country could be related to strains from the same or different farms. The 627 *E. coli* isolates comprised of 182 different sequence types (ST) types with 24 isolates being of unknown ST type, indicating high diversity in the *E. coli* isolates. The major identified ST type was ST10 (n = 143, 23%) and *E. coli* isolates seems to be clustered based on ST type rather than geographical location, animal reservoir, *E. coli* pathotype or AMR (Fig. 1). Similar tree topology of the *E. coli* isolates was observed in core genome MLST tree (Fig. S11).

**Figure 2:**
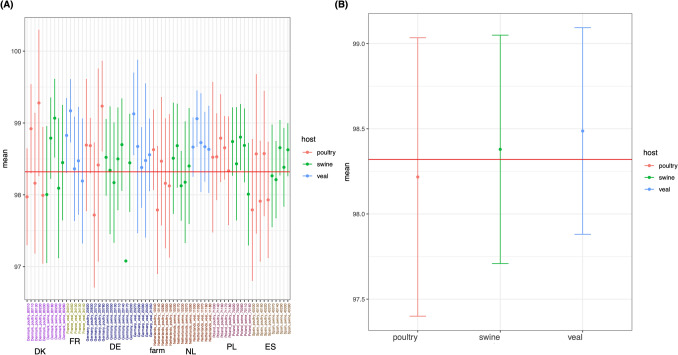
Average nucleotide identity in mean percent identity of *E. coli* isolates from the same farm (**A**) and from the same animal host (**B**). *DK* Denmark, *FR* France, *DE* Germany, *NL* The Netherlands, *PL* Poland and *ES* Spain. Vertical line represents points within 1st/3rd + 1.5 IQR. A red horizontal line indicates mean of overall nucleotide identity (98.34%).

Average nucleotide identity (ANI) from comparative genomic of the *E. coli* isolates was summarised on farm and animal host levels (Fig. 2). Mean of overall ANI was 98.3% (red horizontal line). Five out of 10 *E. coli* isolates from Danish and Spanish farms and five of 15 isolates from Netherlands had ANI lower than average (98.3%). Meanwhile Germany, France and Poland had three out of 17, one out of five and one out of 10 farms with ANI lower than average respectively. Most of the lower average ANI farms were poultry and swine. Figure 2B showed that *E. coli* isolates from poultry had the lowest mean ANI and broadest range of ANI compared to the ones from swine and veal, reflecting the high strain diversity.

### Antimicrobial resistance genes

**Figure 3:**
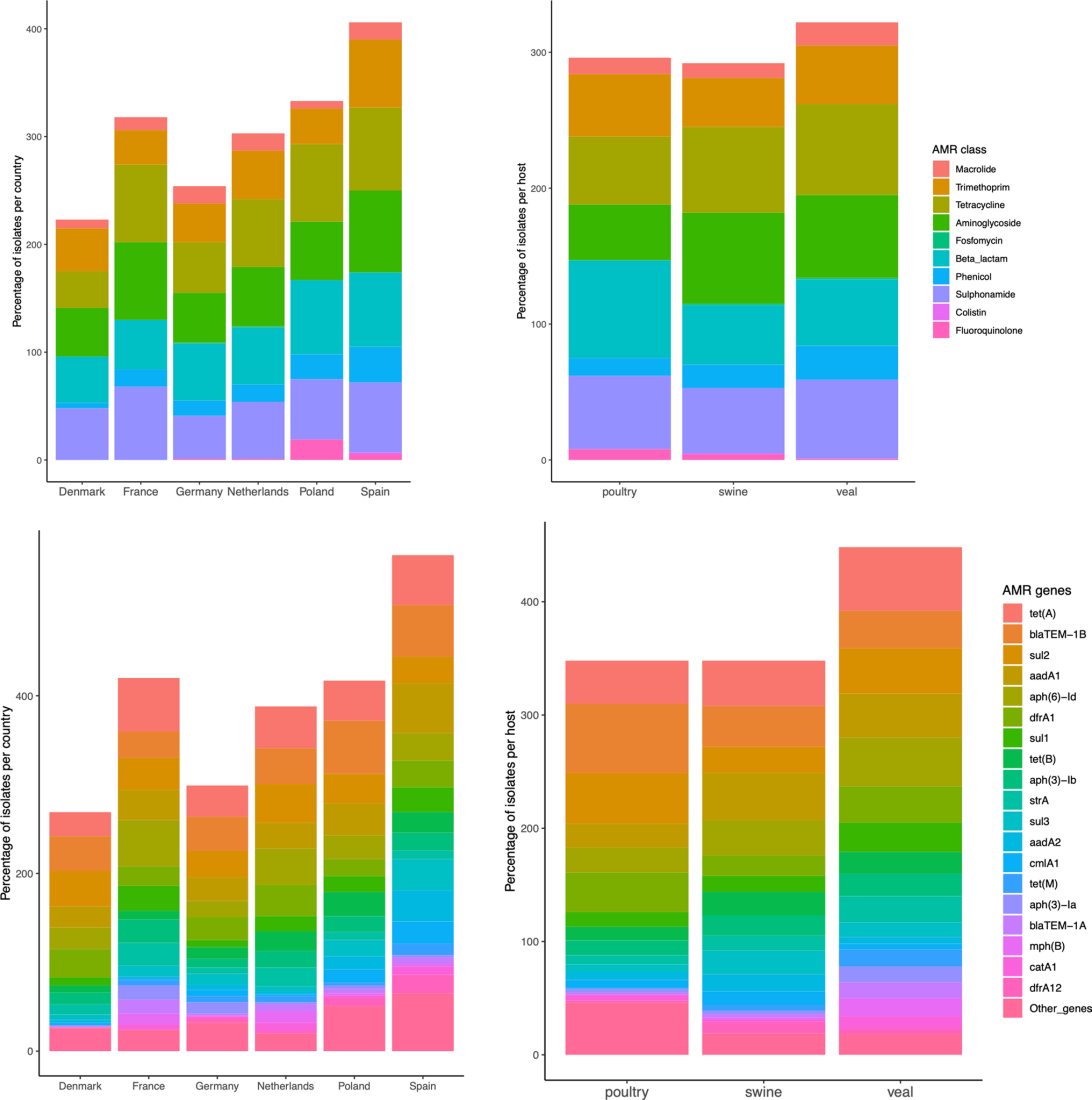
Bar plot showing percentage of resistant *E. coli* isolates in AMR classes (top) and genes (bottom) stratified by country (left) and host (right).

**Figure 4:**
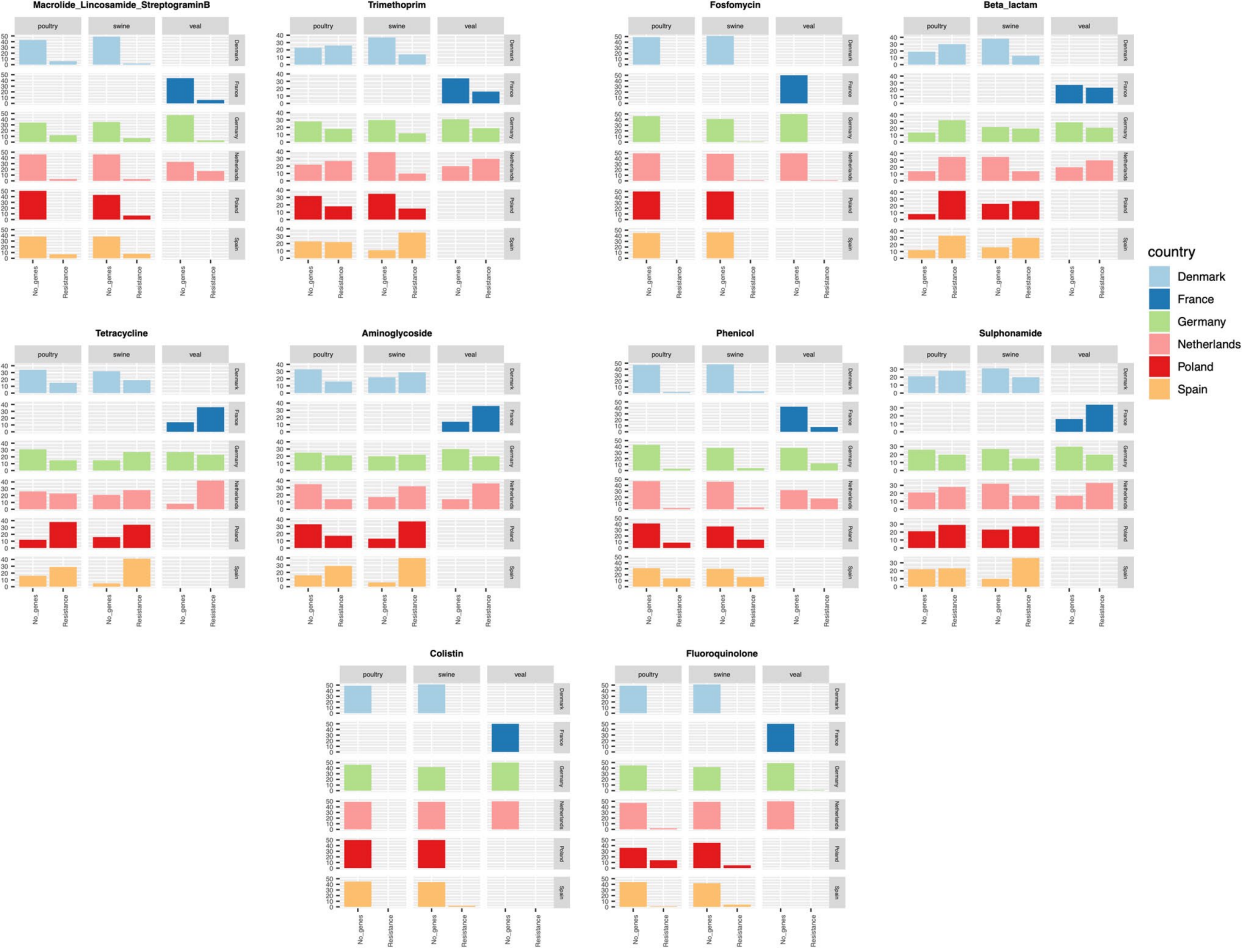
Distribution of resistance and susceptible *E. coli* isolates in different AMR class and host. Y-axis is number of isolates. Three columns are number of isolates from poultry, swine and veal. In each column, first bar is number of susceptible isolates and second bar is number of resistant isolates.

Prevalence and distribution of resistant *E. coli* strains per country and host were illustrated in Figs. 3 and 4. Denmark had the lowest abundance of AMR strains, meanwhile Spain had the highest abundance. All countries had a similar distribution of AMR with the exception of some genes that were more abundant in Poland, Spain and France. Poland had higher proportion of fluoroquinolone resistant *E. coli* (Figs. 3, 4) which were caused by *qnr*B19 and *qnr*S1 genes (Fig. S12). Spain and France had higher proportion of sulphonamide resistant *E. coli* (Figs. 3, 4) associated with *sul1, sul2* and *sul3* genes (Fig. S12 and Table S1 sub-sheet “Percentage of resistant *E. coli*”). Regarding the animal reservoir, isolates from veal calves had higher proportion of AMR resistant *E. coli* strains (Fig. 3), while the proportion of fluoroquinolone resistant *E. coli* was higher in poultry and swine. Additionally, the proportion of beta-lactam resistant *E. coli* was higher in poultry which was caused mainly by *bla*_TEM-1B_ (Fig. S12 and Table S1 sub-sheet “Percentage of resistant *E. coli*”).

The prevalence of AMR was ranked based on percentage of *E. coli* carrying AMR genes in country of origin and animal host. The most prevalent five AMR genes were revealed (Table S1 sub-sheet “Percentage of resistant *E. coli*”). They were *tet*(A), *bla*_TEM-1B_, *sul*2, *aad*A1 and *aph*(6)-Id coding for resistance against tetracyclines, aminopenicillins, sulphonamides and aminoglycosides. These five AMR genes were also the top five AMR genes in the strains from France and Poland. Three to Four of them were among the top five AMR genes in other countries in this study. In addition, these five AMR genes were most prevalent AMR genes in the *E. coli* strains from swine and veal calves. Four of them were among the top five AMR genes in poultry (Table S1 sub-sheet “Percentage of resistant *E. coli*”).

The most prevalent five AMR genes were analysed for conserved regions on 1 kb upstream and 1 kb downstream of the gene (Fig. S13). Although, upstream and downstream regions from almost all five AMR genes showed variable regions and not any large conserved regions, the upstream region from *aph(6)-Id* exhibited ~ 600 bp conserved region among the *E. coli* isolates (Fig. S13, grey colour) suggesting that among the top 5 AMR genes, only *aph(6)-Id* gene seems to be specifically conserved in *E. coli* isolates.

### Mobile genetic element linked with plasmid, AMR and virulence genes

**Figure 5:**
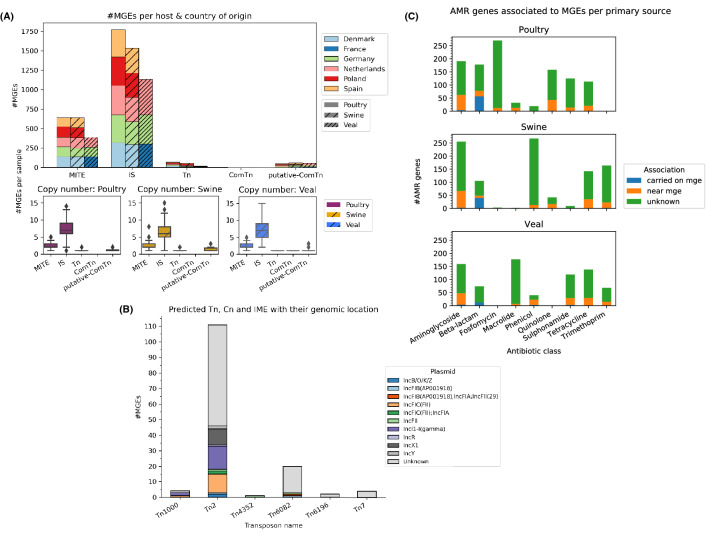
(**A**) Distribution of mobile genetic elements (MGEs) in *E. coli* isolates per animal host and country of origin (top). Distribution of MGEs per *E. coli* isolate in different animal hosts (bottom). *Cn* XX, *IS* insertion sequences, *MITE* miniature inverted-repeat transposable elements, and *Tn* transposons. (**B**) Distribution of transposons and their plasmids. (**C**) Number of MGEs associated with mobile elements per antimicrobial class and animal source. There are three levels of associations, carried on MGEs, if MGE is located within 31 kbp from an AMR and unknown association.

There was little difference in both total number of MGEs and in the average number of MGEs carried by an *E. coli* between the different animal hosts (Fig. 5A). However, the composition of MGEs carried by *E. coli* differed between the hosts. More unity transposon (Tn) were predicted in isolates from poultry and swine than in isolates from veal (Fig. S14). Some insertion sequences (IS) were very prevalent, for instance ISEc*1* and IS*609* were found in 87.9% and 79.9% of all isolates (Table S2). The predicted IS existed in multiple copies with varying degree of alignment completeness and level of truncation compared to the reference sequence. For all of the predicted IS a majority of the identified copies were highly truncated but had an intact inverted repeat region which could help form composite transposons (ComTn) (Tables S2 and S3). The most frequent transposon was Tn2 (Fig. 5B) which was frequently found on IncX1, Incl1-I(gamma) and IncFIC(FII) plasmids (Fig. 5B). Tn*2* is known that *E. coil* unit-transposon carries *bla*TEM-1 gene^11^.

*Escherichia coli* isolates were clustered on the predicted MGE profile using Jaccard distance and complete linkage for all strains (Fig. S15) and isolates per country (Figs. S16–S21). The trees showed the distribution of MGEs within the *E. coli* population and any relationship with country, animal source, farm ID or ST type. *Escherichia coli* isolates carried a highly diverse set of MGEs that were seemingly independent of host or ST type. These *E. coli* isolates carried on average ~ 4 plasmids but a minority of the predicted MGEs were located on them (Fig. S22).

AMR gene was classified based on their location in respect to MGEs. A gene considered to be associated if it was located closer than 31 kbp to an MGE, mobilized if it was an accessory gene within the MGE, and having an unknow association if neither criterion were met. A gene associated with an MGE could be mobilized through formation of ComTn. Beta- lactamase type AMR genes were more frequently mobilized by MGEs more than genes from other AMR classes. In addition, more beta-lactamases were carried on transposons in poultry and swine than in veal calves (Fig. 5C). These carried beta-lactamases were mainly *bla*_TEM-1_ carried on transposon Tn*2*. In addition, three *tet*(A) genes were mobilized on Tn*1721* (Table S4). Some putative ComTn were identified where the most abundant mobilized a variety of AMR genes (Table S5).

**Figure 6:**
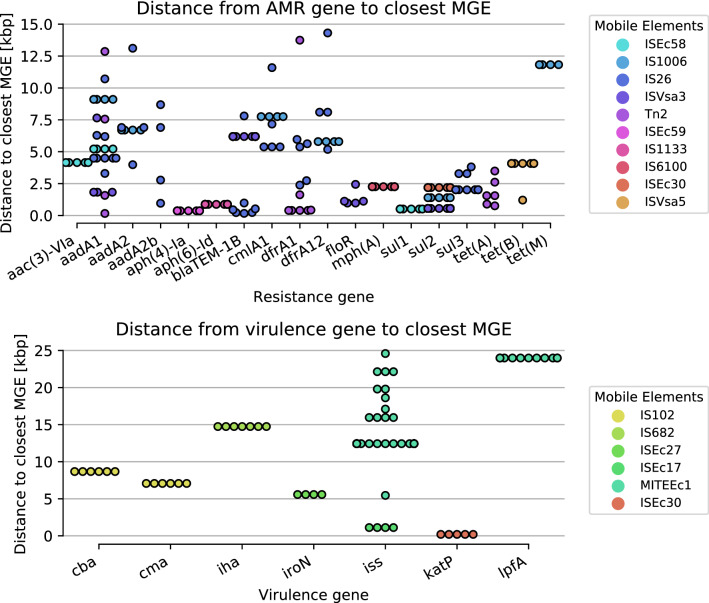
Distance from AMR gene (top) and virulence gene (bottom) to closest MGE.

The distance between the gene and the MGE its associated with were shown in Fig. 6. MGEs such as IS*26*, ISVsa*3* and Tn*2* were associated with several AMR genes where IS*26* was associated with the most MGEs (Fig. 6). MGEs tended to be located at a fixed distance from a gene. This could be an indication that they are transferred as a unit. One indication that the AMR genes was mobilized by the MGEs are their tendency to be located arrays of repeated genes with variable length and content. These arrays have MGE located up to 4151 bp downstream of the first AMR (Table S6). However due to the low number of observations these results are rather speculative.

### Abundance of AMR from metagenomic and whole genome sequencing

**Figure 7:**
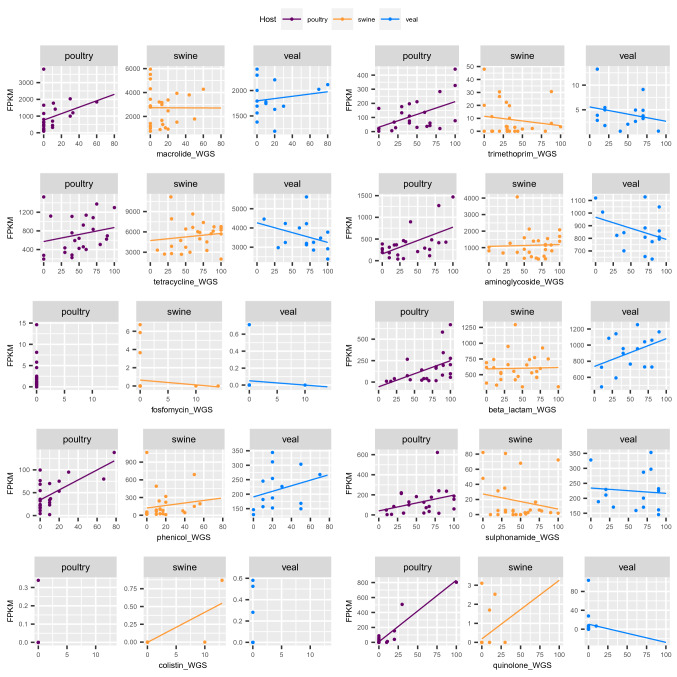
Scatter plot between abundance of AMR genes from metagenomic fecal samples and percentage of *E. coli* isolates carrying AMR genes from the same farm. X-axis is percentage of *E. coli* isolates carrying resistance genes. Y-axis represents abundance of resistance genes in FPKM (Fragments Per Kilo base per Million fragments) from metagenomic fecal samples.

Fecal samples used for *E. coli* isolation were sequenced using shotgun metagenomics^8^ and AMR genes were characterized in the metagenomics data using the ResFinder database. Abundance of AMR genes from metagenomic fecal samples and *E. coli* whole genome sequencing from the same farm was illustrated as scatter plot in Fig. 7. The AMR abundance in the metagenomic samples were calculated as fragments per kilobase reference per million bacterial fragments (FPKM), while the AMR abundance in *E. coli* WGS was determined as percentage of *E. coli* isolates carrying AMR genes. Correlation and *p* value from the regression analysis in Fig. 7 was shown in Table 1. There was significant correlation between AMR abundance of metagenomics and AMR prevalence in *E. coli* WGS from poultry in trimethoprim, aminoglycoside, beta-lactam, phenicol and quinolone (5 out of 8 AMR classes present in *E. coli* WGS from poultry). Correlation between AMR abundance in colistin was found only in swine samples. Significant correlation was not observed in veal samples. This suggested that AMR abundance from poultry feces could potentially represent AMR abundance in single isolation of *E. coli* from poultry.

**Table 1:** Correlation and *p* value from regression analysis between abundance of AMR genes in metagenomic fecal samples and percentage of *E. coli* isolates carrying AMR genes.

AMR	Poultry		Swine		Veal	
	Correlation	*p* value	Correlation	*p* value	Correlation	*p* value
Macroline	0.35	0.099	− 0.0034	0.99	0.17	0.55
Trimethoprim	**0.52**	**0.0099**	− 0.16	0.43	− 0.24	0.38
Tetracycline	0.22	0.31	0.14	0.5	− 0.34	0.22
Aminoglycoside	**0.55**	**0.0053**	0.039	0.84	− 0.34	0.21
Fosfomycin	–	–	− 0.096	0.64	− 0.071	0.8
Beta lactam	**0.49**	**0.014**	0.029	0.89	0.4	0.14
Phenicol	**0.65**	**0.00056**	0.15	0.46	0.29	0.3
Sulphonamide	0.34	0.1	− 0.2	0.32	− 0.089	0.75
Colistin	–	–	**0.78**	**1.3e−06**	–	–
Quinolone	**0.93**	**4.7e−11**	0.27	0.18	− 0.037	0.9

The bold value represents significant correlation between the abundance of AMR genes from metagenomic samples and single isolation.

## Discussion

This study is a large-scale multinational study of *E. coli* isolates from several animal sources within the same year. The 627 *E. coli* exhibited a considerable degree of genetic diversity, differing by a total of 17,213 SNPs and representing 182 different ST types. A recent study on genetic diversification from a large collection of non-clinical *E. coli* isolates also revealed 442 distinct ST types of which 61% were from a single strain^12^. *Escherichia coli* strains isolated from humans, domesticated and wild animals represent a primary habitat of *E. coli*^5^. Most of the *E. coli* from these domesticated animals were not harmful pathogenic strains and 6% were identified as EAEC, ETEC or STEC. The high genetic diversity in the *E. coli* isolates resulted in phylogeny which showed no clustering based on country of origin, animal reservoir and antimicrobial resistance. Moreover, the strains within the same country could be more related to strains from different farms. The results suggested that the *E. coli* strains could be randomly spread between the farms in each country. A recent study on *E. coli* isolates collected from pig surveillance between 2013 and 2017 in the United Kingdom also found no correlation between phylogeny and year of isolation^13^. This suggests that *E. coli* isolates could be as well randomly spread in countries and through different years. An alternate explanation would be that the *E. coli* we have sequenced do not fully represent the extent of the diversity within each animal, farm, and country.

Average nucleotide identity (ANI) is a measure of genomic divergence between genomes. The ANI of *E. coli* isolates from different countries did not exhibit any clear differences among countries. Nonetheless, *E. coli* isolates from poultry showed lowest and broadest range of ANI suggesting that the poultry *E. coli* had higher genetic diversity and were more heterogenous than isolates from swine and veal calves. Previous study concluded that *E. coli* genotypes and serotypes in pullets and layers are heterogeneous and do not maintain a single clonality within the same bird^14^. In addition, *E. coli* do not show definite clonal population structure based on geographical region and age of the host^14^. In animals, the main environmental force shaping the genetic structure of *E. coli* gut population is domestication status of the host^15^ and socioeconomic factors, such as diet^16^ and hygiene, are presumably the main factors accounting for phylogenetic group distribution rather than geographical or host genetic conditions^5^.

According to phylogenetic result and distribution of AMR genes in *E. coli* from different countries, we suggested that AMR seems to spread randomly in all the surveyed countries. Exceptions were higher fluoroquinolone resistance in Polish strains and higher sulphonamide resistant *E. coli* in Spain and France. Veal calves had a higher proportion of resistant *E. coli* strains. A previous study on commensal *E. coli* from a systematic resistance monitoring of primary food production, slaughterhouses and retail, they found that dairy cows had the lowest number of resistant *E. coli*, whereas veal and pork had higher frequency of resistance strains ranging between 43 and 73%^17^. In addition, our results showed that fluoroquinolone and beta-lactam resistant *E. coli* was higher in poultry. Similar results were found in a study in Czech Republic on *E. coli* from poultry meat, poultry farms and market-weight turkeys. They found that resistance of the poultry isolates to quinolones ranged from 53 to 73%^18^. Another study on *E. coli* from broilers, workers who worked in poultry slaughterhouses and clinical specimens in Romania, indicated a very high rate of ESBL and AmpC-producing *E. coli* in chickens^19^.

Genes are mobilized through the interplay of several MGEs of different types. Elements with intra-cellular transposition, such as IS and Tn, can mobilize new genes and transport them to conjugative/ mobilizable transposons and plasmids^10^. AMR genes associated with integrative MGEs (iMGEs) tended to be located at fixed distances from the iMGE, and was often part of an array of AMR genes. Some arrays exhibited greater variability of the genes contained the arrays than other. This indicates that these genes could be part of an integron, which in turn could be mobilized by the associated IS through the formation of a ComTn. Of these arrays the *aph(6)-Id* associated with IS*1133* and *mph(A)* associated with IS*6100* seemed to have less variation in the genes compared to the other arrays. The lack of variation might be caused by the associated MGEs are less prone to form ComTn.

The distribution of MGEs within the *E. coli* isolates revealed the lack of well-defined clusters and comparably dissimilar profiles suggesting that *E. coli* has a diverse and flexible mobilome. This could mean that mobile genetic elements are more mobile in *E. coli*, either due being frequently mobilized on plasmids or higher conjugation rate. Plasmids are important vehicles for the carriage of MGEs and AMR genes^10^, the result showed that the *E. coli* carried approximately four plasmids on average but a minority of the MGEs were located on them. However, the number of mobilized MGEs are likely underestimated due to a combination of the limitations in the detection method of PlasmidFinder^20^ and the difficulty assembling repetitive DNA, and with that plasmids, from short read sequence data. Moreover, a significant number of plasmid sequences in INSDC databases (DDBJ, EMBL-EBI, NCBI) may be misassembled, as evidenced by the presence of fragments of MGEs that are not explained by truncation by other MGEs^21^. To further study the mobility and distribution of AMRs one would need to construct near complete the assemblies, preferably via hybrid assembly using long read sequencing as a template, and combine it with additional plasmid prediction tools that do bases it predictions on circular features in the data.

When looking at combinations of MGE, distances and AMR genes that were observed more than four times they were primarily found on a single type of plasmid which could explain the observed pattern.

MGEs can influence the expression and/or mobility of nearby genes through a variety of mechanisms. Through the formation of ComTn can an IS, which lacks accessory genes, transpose several gens as unit within the cell. We identified several putative composite transposons carrying AMR genes based on the location and completeness of IS elements. Of these were IS*26* based elements both the smallest, only carrying one or two genes, and the one most frequently predicted to carry different genes. This is likely caused by the tendency of IS*26* to form arrays of repeated MGEs^22^. However, further research is needed to verify if the putative ComTn can mobilize these genes.

*AMR* genes that were found within 31 kbp of a MGE was considered to be associated, meaning that it has the potential to be mobilized. The threshold corresponds to the length of the largest ComTn from *Enterobacteriacea* in MobileElementFinders database (Tn*6108*). Association between MGEs and AMR genes showed that most of the AMR genes carried on MGEs were from beta-lactam class especially *bla*_TEM-1_ and beta-lactamases were mainly carried on transposons in poultry and swine. In addition, MGEs such as IS*26*, ISVsa*3* and Tn*2* were associated with many AMR genes. IS*26* has played a crucial role in the dissemination of resistance determinants in Gram-negative bacteria^10,22^. AMR genes such as *bla*_TEM_ genes, including extended-spectrum beta-lactamases (ESBL) encoding genes have been known that they have been found within Tn*1*, Tn*2* and Tn*3* transposons^10^. Analysis of the distance from AMR and virulence genes to the nearest MGEs indicated that MGEs tended to be located at a fixed distance from the AMR and virulence genes suggesting that the AMR and virulence genes in the *E. coli* isolates have been in part mobilized by the associated MGEs.

The predicted MGEs were only of types that are comparably and performs intra cellular transposition. We did not identify any conjugative/mobilizable transposons such as SXT or ICE*Ec2*^10^ suggests that we are missing these classes MGEs which play and important for the mobilization of genes. These elements are likely not being predicted due to the difficulty in assembling these elements with short read data. Like plasmids, are these elements large and contains repetitive sequences and other MGEs which could confuse the assembler resulting in fragmented assemblies. To overcome these shortcomings, one would likely need to employ long read sequencing. While phages play an important role movement of AMR genes they have not been addressed in this work.

Comparing abundance of AMR from metagenomic analysis of fecal samples and whole genome sequencing of *E. coli* isolated from the same fecal samples, it showed that 5 main AMR classes out of 10 analyzed classes exhibited significant correlation of AMR abundance between metagenomics and WGS results in *E. coli* poultry isolates. No significant correlation of AMR abundance was found in any AMR classes between metagenomics and WGS analysis in swine (except colistin) and veal calves. This result suggested that metagenomics analysis could be used as a predictor for abundance of AMR from bacteria isolates in poultry.

## Conclusion

Commensal *E. coli* isolates from different farms in different European countries in this study indicated neither clonality nor clustering based on geographical location of countries and genetic markers such as antimicrobial resistance and virulence genes. The *E. coli* tended to sporadically spread internationally. Nonetheless, mobile genetic elements play a role in the acquisition of antimicrobial resistance and virulence genes. Abundance of AMR was agreeable between metagenomic and whole genome sequencing analysis in several AMR classes in poultry isolates.

## Materials and methods

### Selection and collection of isolates

As part of the European Union-funded EFFORT project (www.effort-against-amr.eu), fecal samples from animal farms were collected in 2014–2015 from Denmark (10 farms), France (5 farms), Germany (17 farms), The Netherlands (15 farms), Poland (10 farms), and Spain (10 farms) and. From each farm, up to 10 *E. coli* isolates were isolated and whole genome sequenced from poultry (n = 239 from 25 farms), swine (n = 238 from 28 farms), and veal (n = 150 from 15 farms). Farms were selected based on a ranking of antimicrobial use (AMU). Distribution of number of *E. coli* isolates and a list of strains including metadata can be found in Fig. S1 and Table S1 respectively. In addition, previously sequenced metagenomic samples (poultry^8^, swine^8^ and veal calf [PRJEB39685]) from the same farm and fecal samples that were used for *E. coli* isolation were included in this study to compare abundance of AMR from metagenomic and whole genome sequence analysis.

### DNA extraction and whole genome sequencing

Genomic DNA was extracted from *E. coli* isolates using PureLink Genomic DNA Mini Kit (Thermo Fisher Scientific), Qiagen, Gentra, or Puregene bacterial kits and DNA concentrations were determined using the Qubit dsDNA BR assay kit (Thermo Fisher Scientific). Sequencing libraries were prepared with the Nextera XT DNA library preparation kit or the Nextera DNA Flex library preparation kit according to the manufacturer’s protocol (Illumina, Inc., San Diego, CA, United States). Paired-end sequencing was performed on the Illumina HiSeq, MiSeq or NextSeq sequencing platform.

Raw sequence data were deposited in the European Nucleotide Archive (http://www.ebi.ac.uk/ena) under study accession no.: PRJEB41365. The raw reads were adapter-trimmed, quality filtered using bbduk (part of the suite bbtools version 36.49)^23^ and de novo assembled using SPAdes version 3.11^24^ Genomic sequence data including ENA accession numbers is available in the Table S1.

### Species identification, conventional seven multilocus sequence typing, screening for AMR genes, chromosomal point mutations, plasmid and mobile genetic elements

Species identification was used to confirm *E. coli* species using KmerFinder 3.2^24–26^. Conventional seven multilocus sequence typing (MLST) was performed using MLST 2.0^27^. Acquired antimicrobial resistance genes as well as chromosomal point mutations causing resistance were determined using ResFinder 4.0^28^. Virulence genes in *E. coli* were predicted using VirulenceFinder 2.0^29^. Plasmid replicon was identified to infer plasmid presence using PlasmidFinder 2.1. Integrated mobile genetic elements were predicted in the assembled genomes using MobileElementFinder version 1.0 with MGEdb v1.0.2^30^. With these tools we could identify plasmids, miniature inverted repeats, insertion sequences, transposons and conjugative transposons. If two complete copies of the same MGE, or one complete copy and one truncated with preserved IR region was predicted within 31 kbp from one another they was flagged as putative composite transposons. In addition was MGEs located within 31 kbp from an AMR or virulence gene was considered to be associated and to have a theoretical ability to mobilize the associated gene^30^. The threshold corresponds to the longest composite transposon in Enterobacteriaceae (Tn*6108*).

#### Average nucleotide identity (ANI)

Average nucleotide identity (ANI) was determined from assembled genomes of *E. coli* strains by all-against-all alignment using FastANI^31^ (https://github.com/kbaseapps/FastANI) to estimate whole-genome similarity among the strains from different countries, animal hosts and farms.

### Single nucleotide polymorphisms tree

Single nucleotide polymorphisms (SNPs) were determined using CSI phylogeny^32^. Basically, paired-end reads were mapped to the reference genome, *Escherichia coli* str. K-12 (accession number; NC_000913.3), using Burrows-Wheeler Aligner (BWA) version 0.7.2^33^. SNPs were identified using ‘mpileup’ module in SAMTools version 0.1.18^34^. Qualified SNPs were chosen using following criteria: (1) a minimum distance of 10 bps between each SNP, (2) a minimum of 10% of the average depth, (3) the mapping quality was above 25, (4) the SNP quality was more than 30, and (5) all INDELs were excluded. The qualified core SNPs were concatenated to a single alignment corresponding to the position of the reference genome. The concatenated SNP sequences were subjected to parsimony tree construction using PhyML^35^ with the HKY85 substitution model.

### *cgMLST* tree

The cgMLST loci sequences were retrieved from Enterobase^36^ (https://enterobase.warwick.ac.uk). Raw reads were mapped with KMA against the cgMLST sequences to identify the best-matched allele numbers. Sequences were indexed with kmer size 16 and threshold for read mapping were match = 1, mismatch = − 2, gap-opening = − 3, gap-extension = − 1 and no threshold for read depth was used^26^. ConClave sorting scheme was used to pick the best matching allele for each cgMLST gene^26^. While all equally well matching alleles were saved in the first iteration, in a second iteration the most likely allele was chosen as the one with the highest number of matches. KMA is freely available at: https://bitbucket.org/genomicepidemiology/kma and https://cge.cbs.dtu.dk/services/kma. The cgMLSTFinder tool, which is based on KMA, can be found at: https://cge.cbs.dtu.dk/services/cgMLSTFinder-1.0/. The pairwise dissimilarities (distances) between genomes were identified based on the allele profile using ‘gower’ distance method from ‘cluster’ package version 2.1.0 in R. The cgMLST trees were constructed from the distance matrix using hierarchical clustering from ‘ape’ package version 5.4-1 in R.

## Supplementary Information

Supplementary Information 1.

Supplementary Information 2.
