# Supplementary material for: Genomic evolution of antimicrobial resistance in Escherichia coli
Source: Sci Rep. 2021 Jul 23;11:15108. doi: 10.1038/s41598-021-93970-7 (PMC8302606; doi:10.1038/s41598-021-93970-7)
Supplement: Supplementary file 2 — Supplementary Information 2. [file 41598_2021_93970_MOESM2_ESM.pdf]

Genomic evolution of antimicrobial resistance in *Escherichia coli*

Pimlapas Leekitcharoenphon[1], Markus Hans Kristofer Johansson[1], Patrick Munk[1], Burkhard Malorny[2], Magdalena Skarżyńska[3], Katharina Wadepohl[4], Gabriel Moyano[5], Ayla Hesp[6], Kees T. Veldman[6], Alex Bossers[7,6], EFFORT Consortium, Magdalena Zając[3], Dariusz Wasyl[3], Pascal Sanders[8], Bruno Gonzalez-Zorn[5], Michael S.M. Brouwer[6], Jaap A. Wagenaar[9], Dick JJ Heederik[7], Dik Mevius[6], Frank M. Aarestrup[1]

1: National Food Institute, Technical University of Denmark, Kgs. Lyngby, 2800, Denmark

2: German Federal Institute for Risk Assessment, Department Biological Safety, Berlin, Germany

3: National Veterinary Research Institute, Puławy, Poland

4: University of Veterinary Medicine Hannover, Bakum, Germany

5. Department of Animal Health and Health Surveillance Center (VISAVET), Complutense University of Madrid, Madrid, Spain.

6: Wageningen Bioveterinary Research, Lelystad, the Netherlands.

7: Institute for Risk Assessment Sciences, Faculty of Veterinary Medicine, Utrecht University, Utrecht, The Netherlands.

8: Fougeres Laboratory, French Agency for Food, Environmental and Occupational Health & Safety, Fougères, France

9: Department of Infectious Diseases and Immunology, Utrecht University, Utrecht.

SUPPLEMENTARY FIGURES

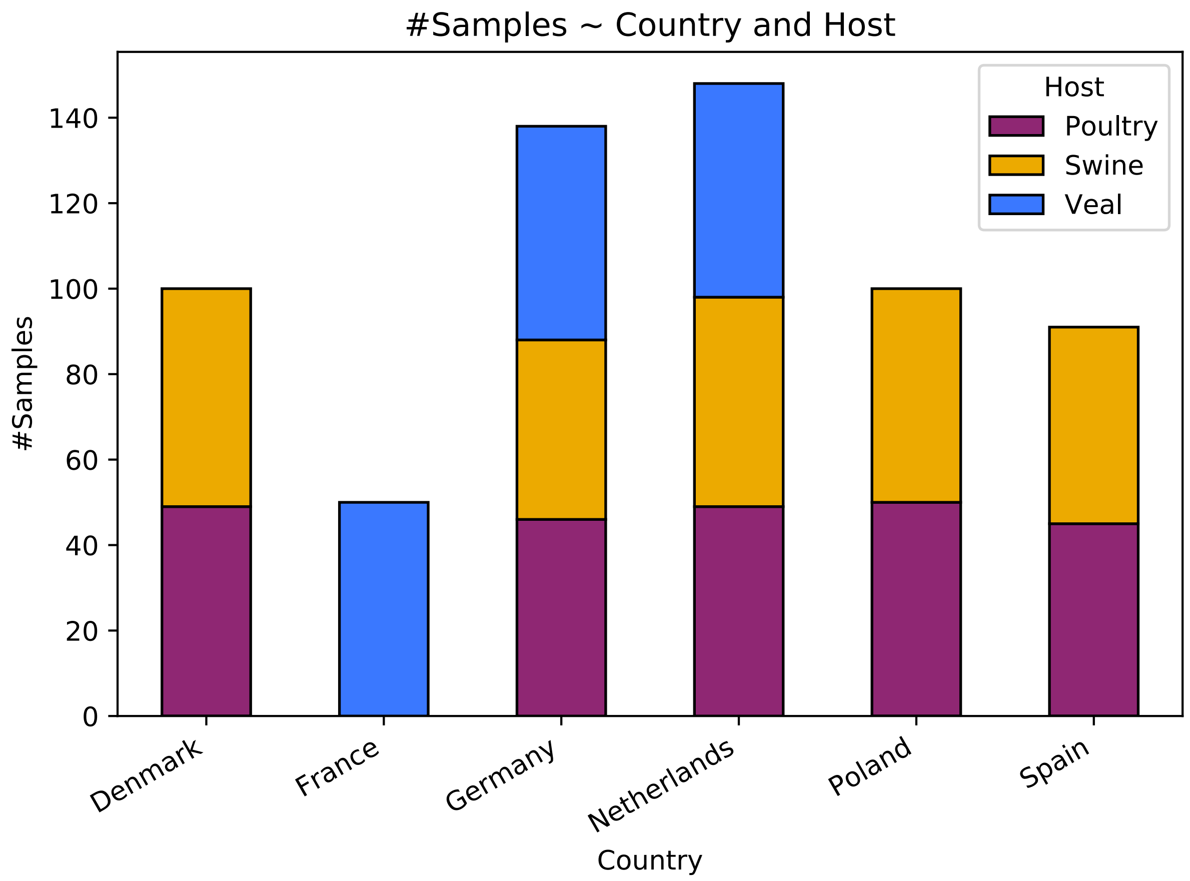

Supplementary Figure 1: Distribution of number of isolates by country and animal host.

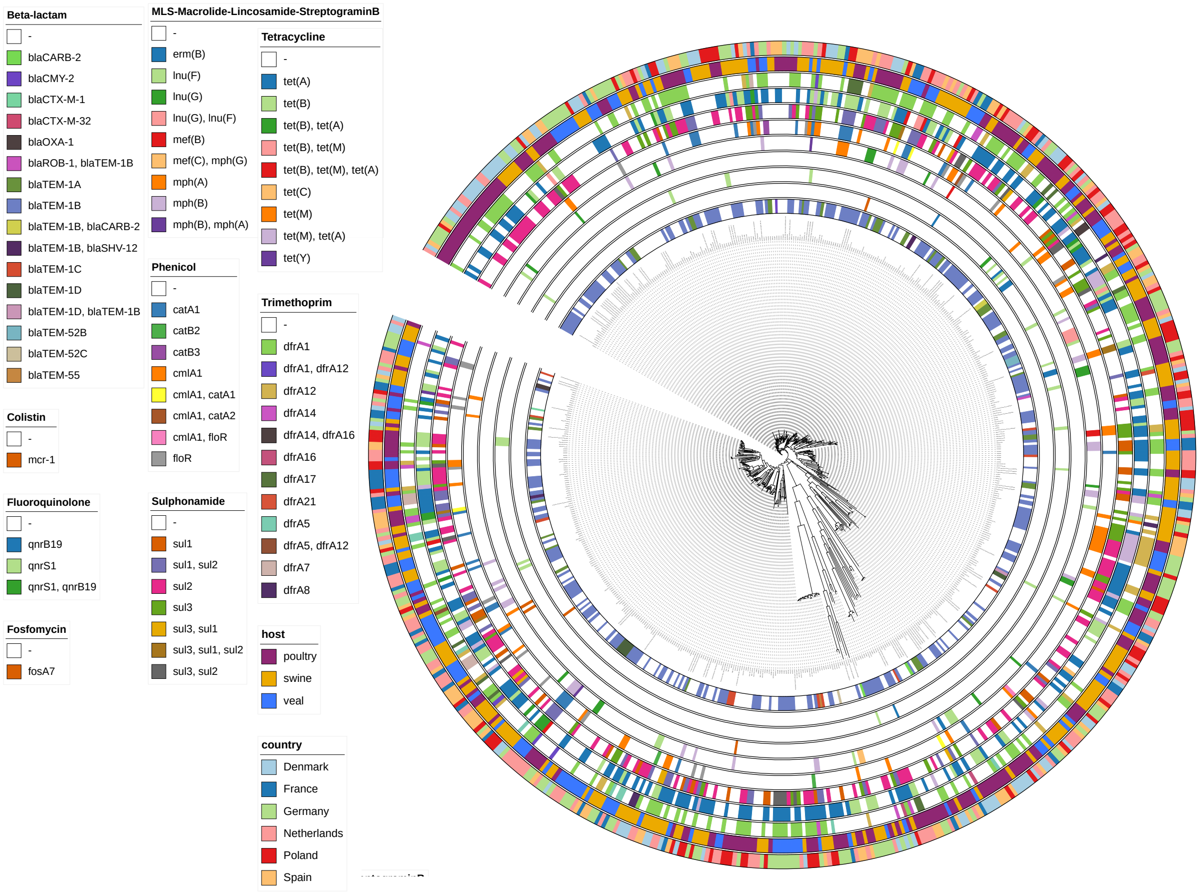

Supplementary Figure 2: SNP tree of 627 *E. coli* isolates with labels of AMR genes from AMR classes except aminoglycoside.

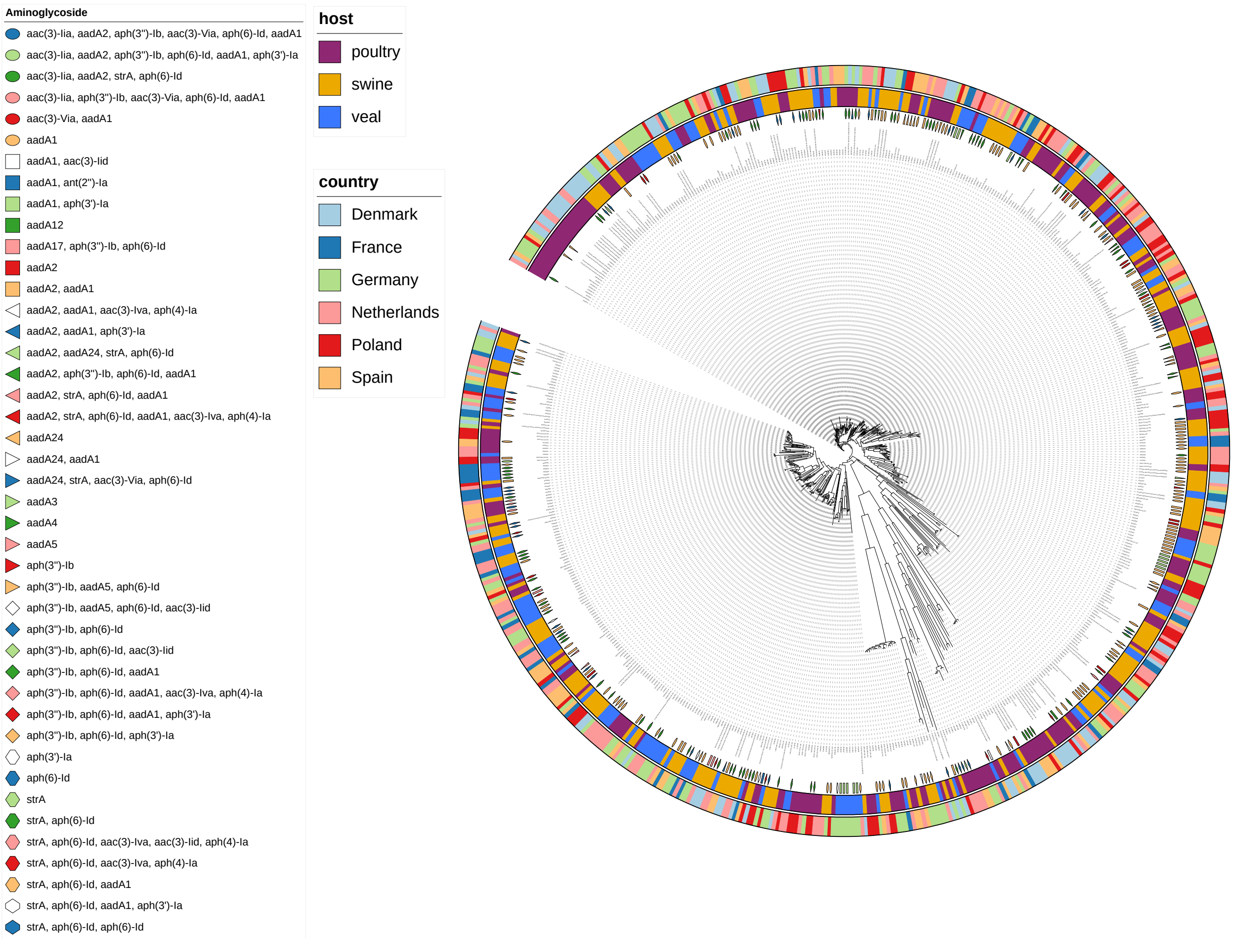

Supplementary Figure 3: SNP tree of 627 *E. coli* isolates with labels of AMR genes from AMR class aminoglycoside.

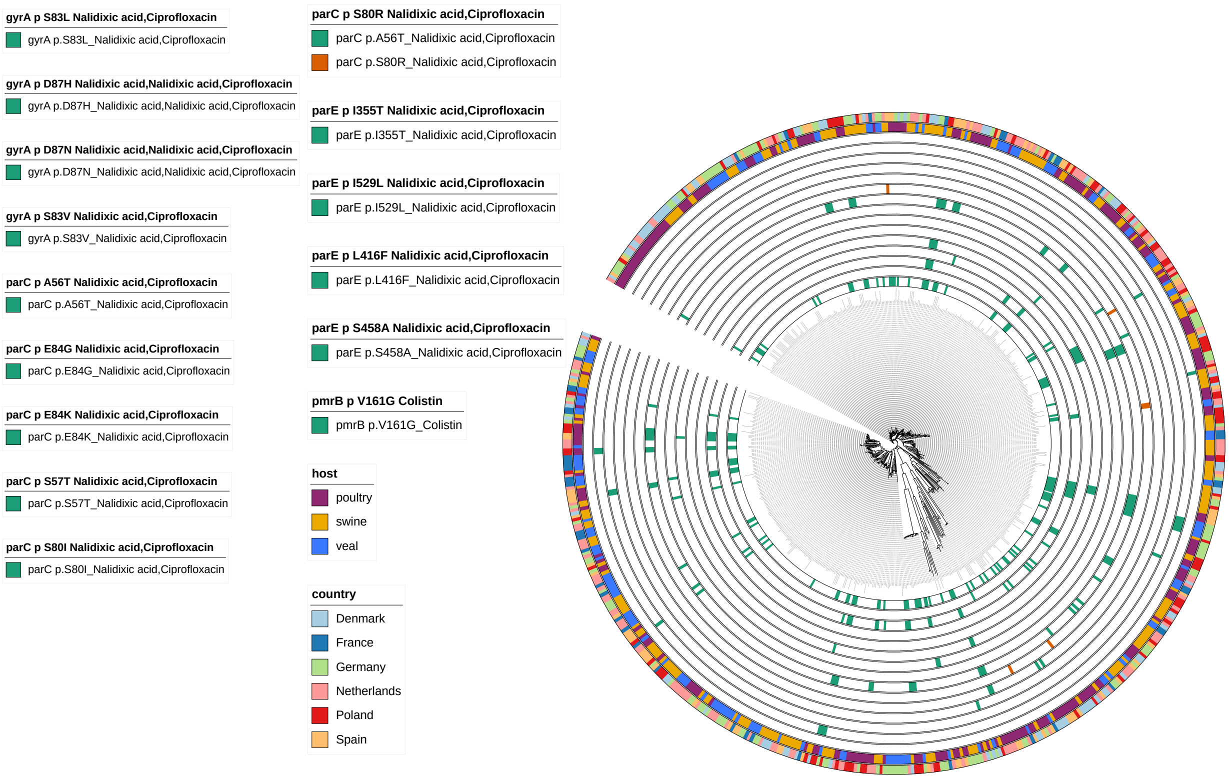

Supplementary Figure 4: SNP tree of 627 *E. coli* isolates with labels of chromosomal point mutation conferring to antimicrobial resistance.

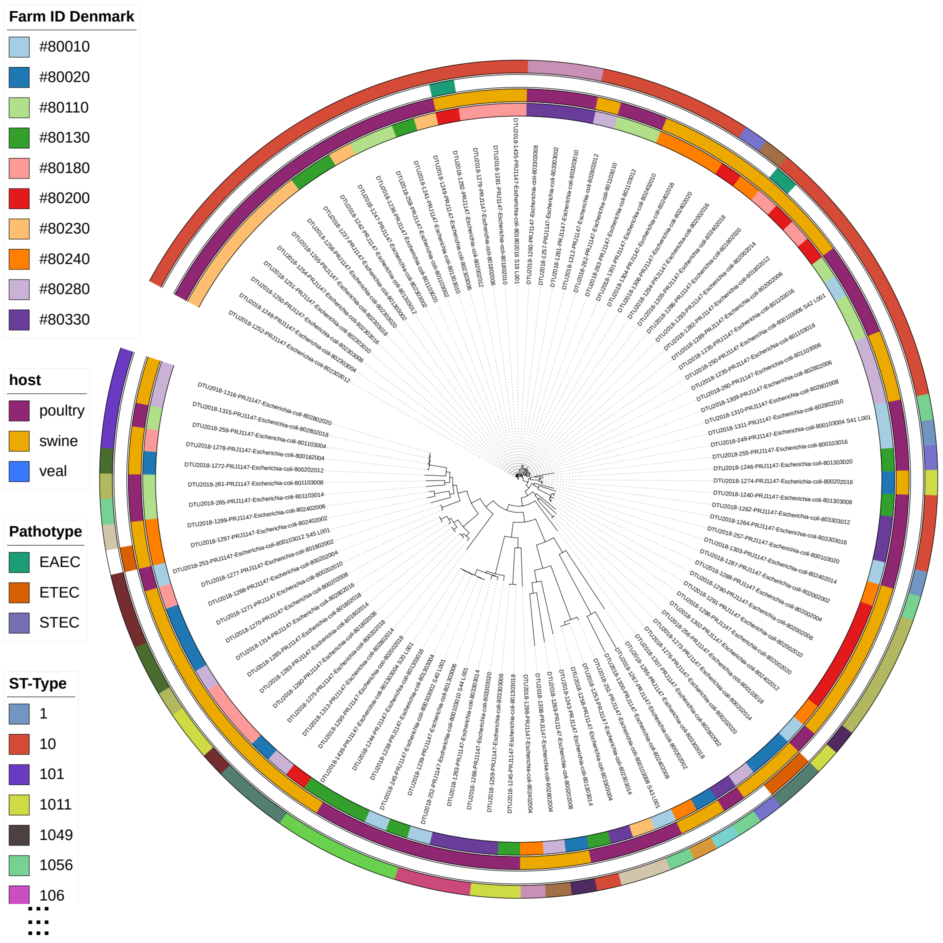

Supplementary Figure 5: SNP tree of *E. coli* isolates from farms in Denmark.

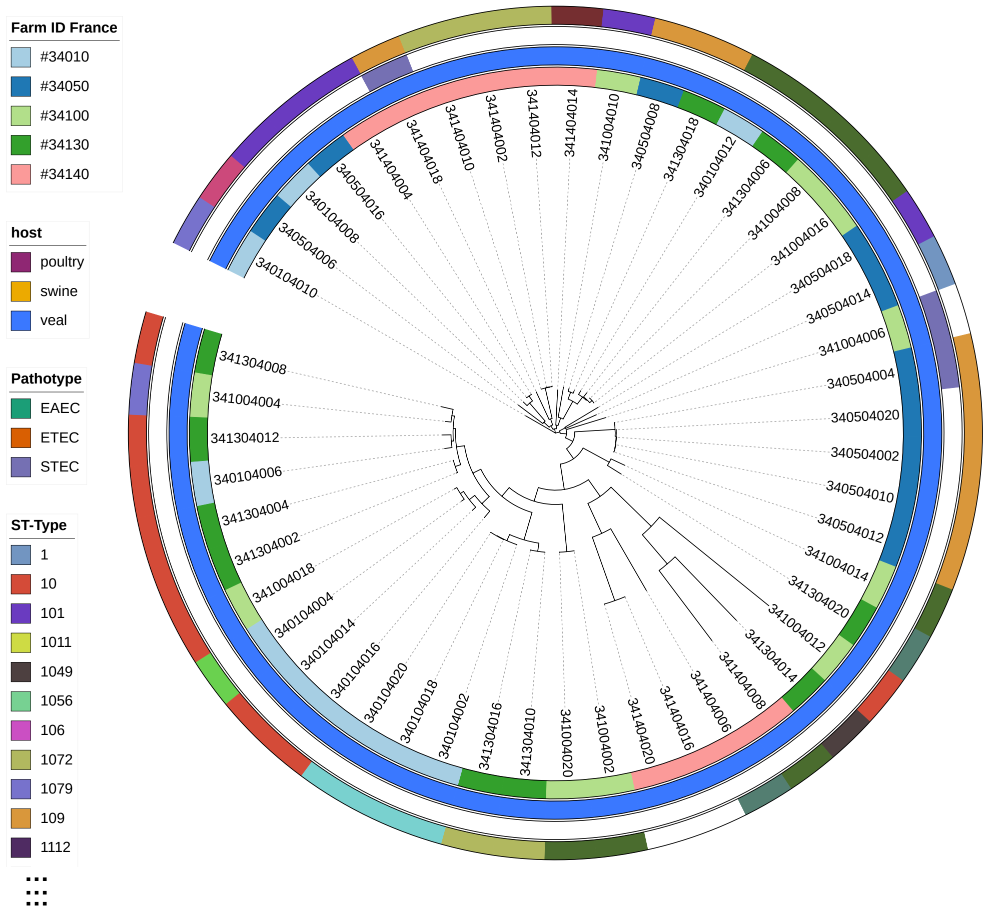

Supplementary Figure 6: SNP tree of *E. coli* isolates from farms in France.

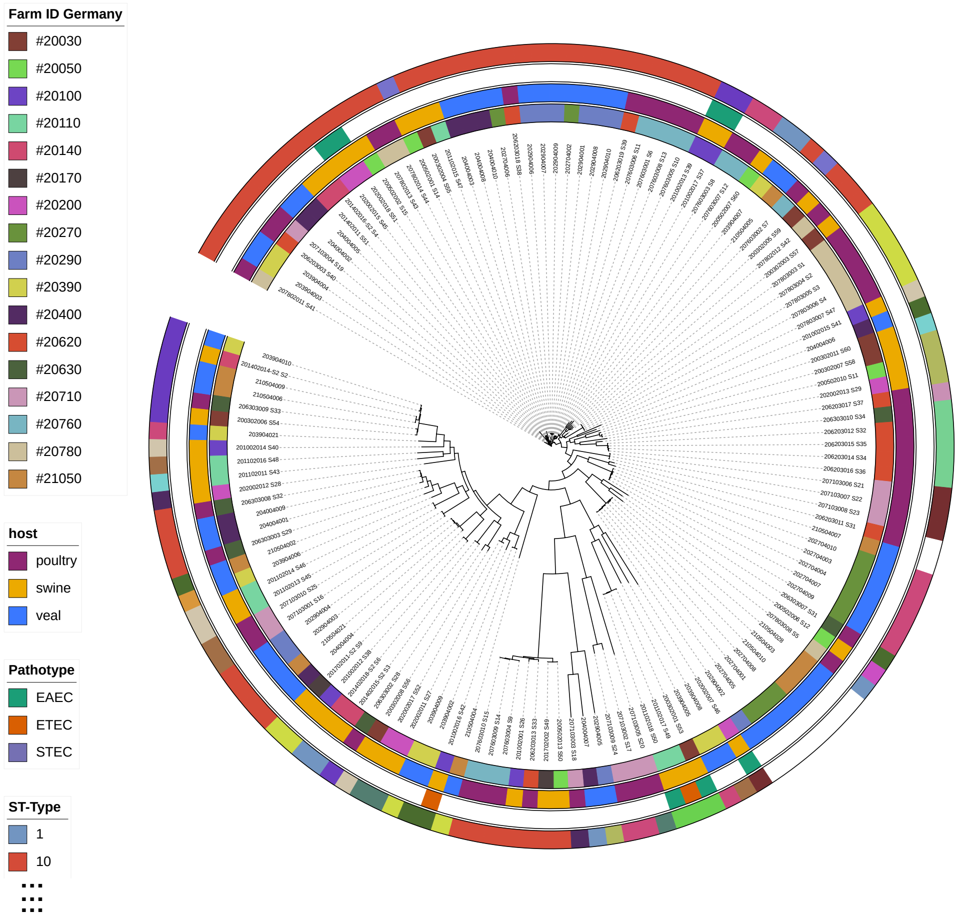

Supplementary Figure 7: SNP tree of *E. coli* isolates from farms in Germany.

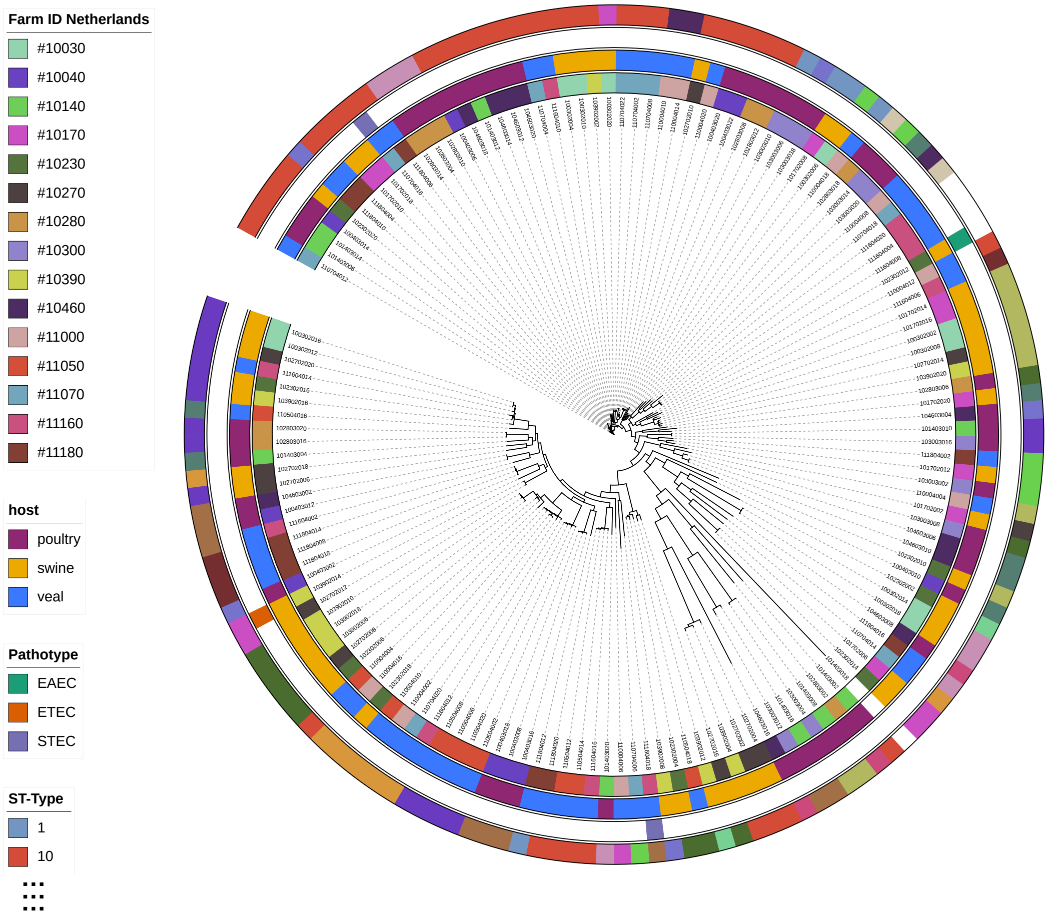

Supplementary Figure 8: SNP tree of *E. coli* isolates from farms in Netherlands.

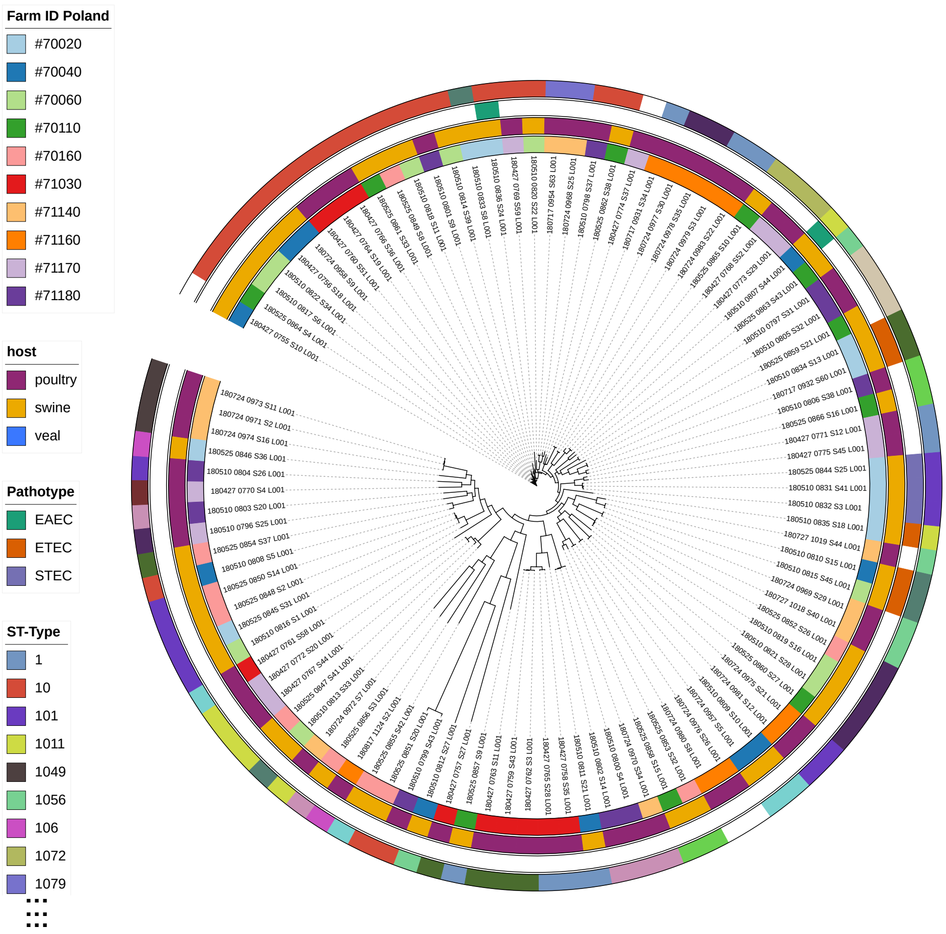

Supplementary Figure 9: SNP tree of *E. coli* isolates from farms in Poland.

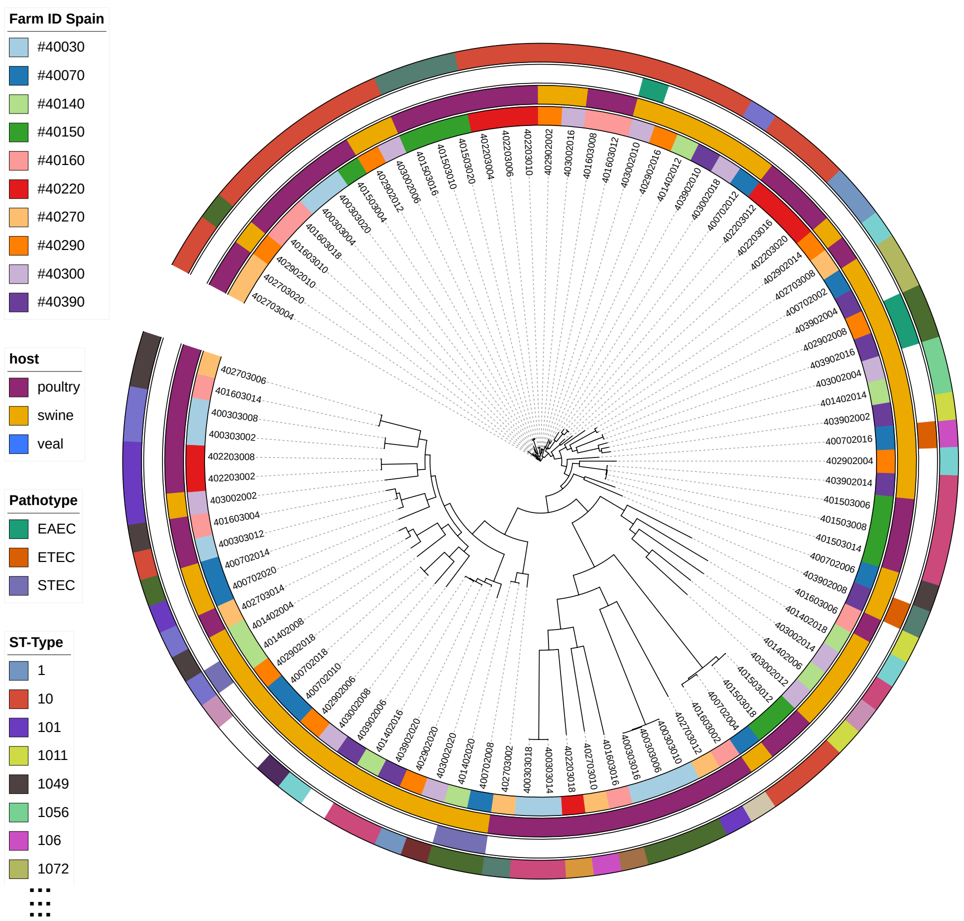

Supplementary Figure 10: SNP tree of *E. coli* isolates from farms in Spain.

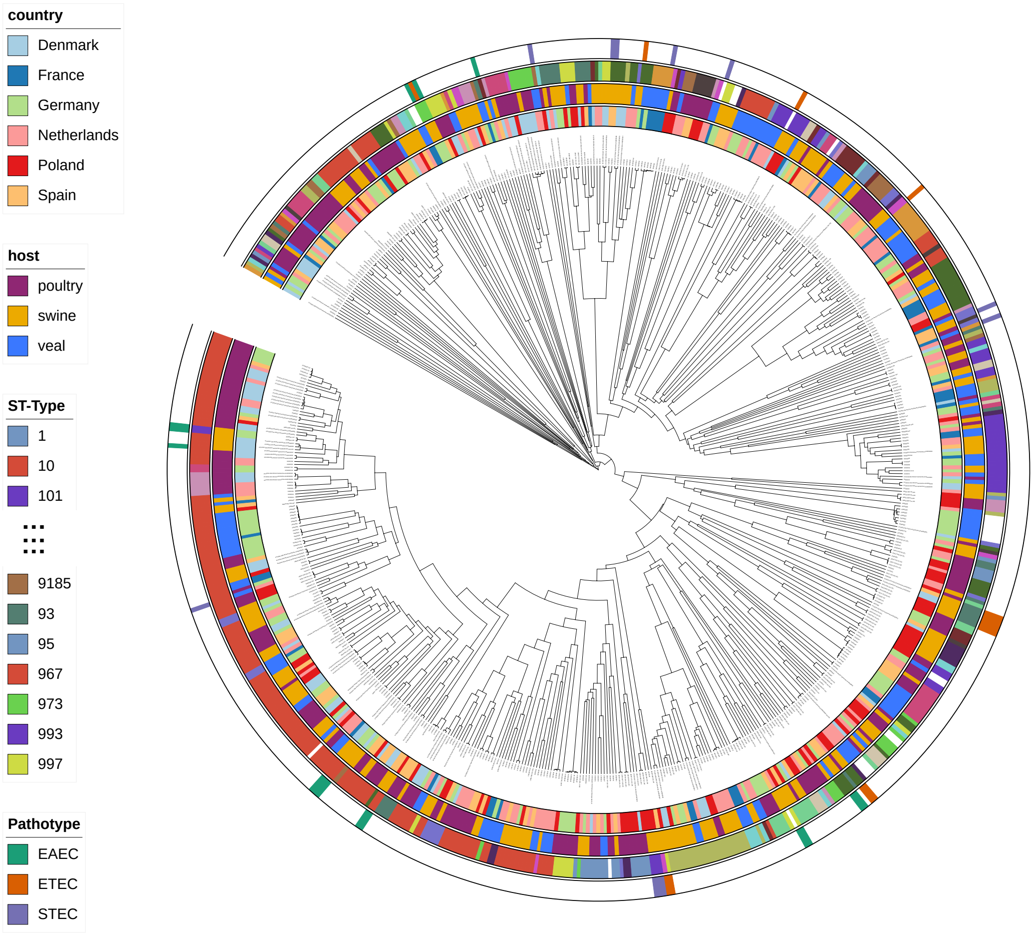

Supplementary Figure 11: cgMLST tree of 627 *E. coli* isolates. The legends from inner circle to outer circle are country, host, ST-Type and pathotype. There are 182 different ST-Type with 24 isolates characterized as unknown ST-Type. Only a number of ST-Type can be showed in figure legend. EAEC: Enteroaggregative *E. coli*, ETEC: Enterotoxigenic *E. coli* and STEC: Shiga toxin-producing *E. coli*.

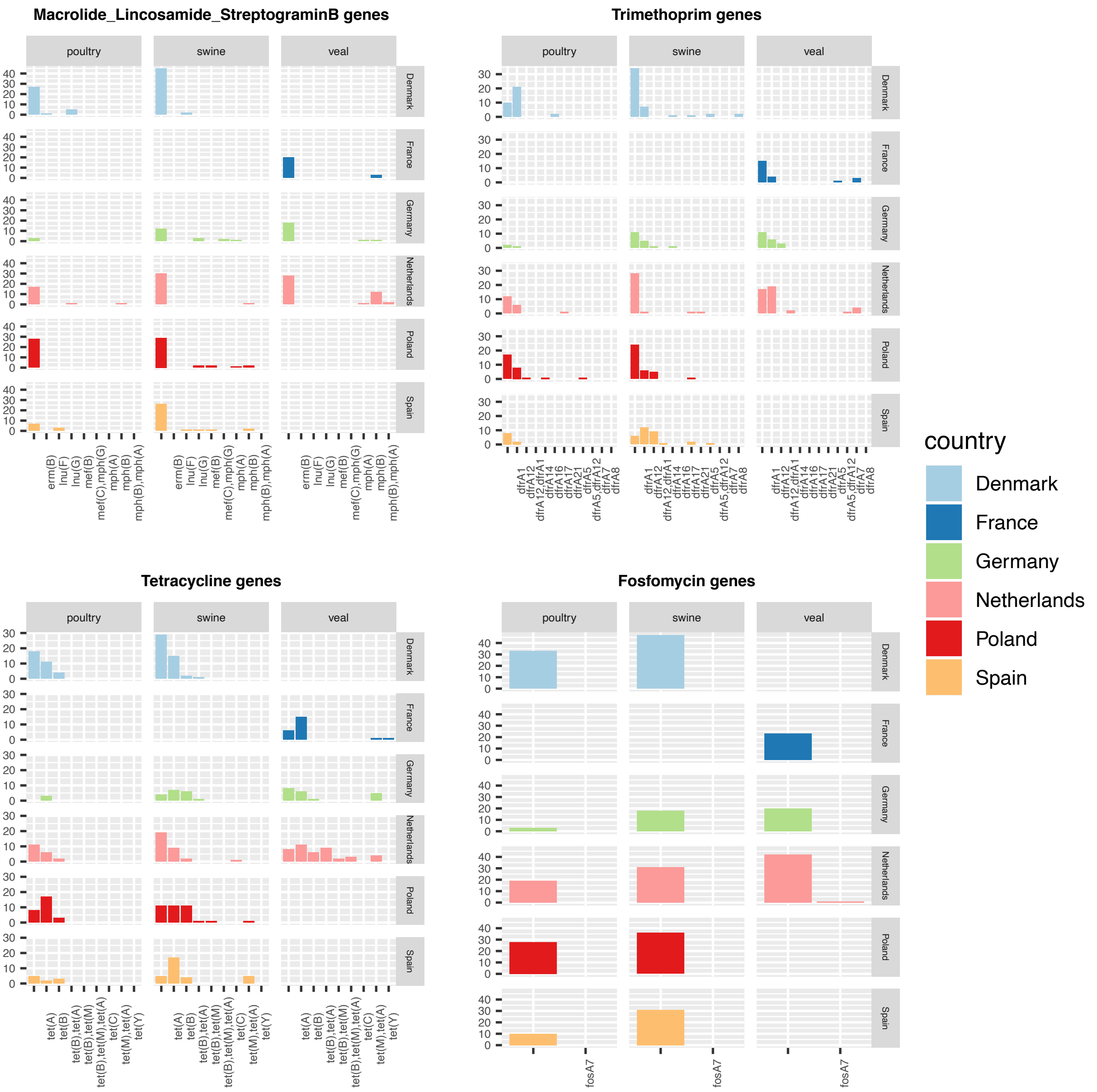

Macrolide_Lincosamide_StreptograminB genes
Trimethoprim genes
Tetracycline genes
Fosfomycin genes
poultry
swine
veal
Denmark
France
Germany
Netherlands
Poland
Spain
erm(B)
lnu(F)
lnu(G)
mef(B)
mef(C),mph(G)
mph(A)
mph(B)
mph(B),mph(A)
dfrA1
dfrA12
dfrA12,dfrA1
dfrA14
dfrA16
dfrA17
dfrA21
dfrA5
dfrA5,dfrA12
dfrA7
dfrA8
tet(A)
tet(B)
tet(B),tet(A)
tet(B),tet(M)
tet(B),tet(M),tet(A)
tet(C)
tet(M),tet(A)
tet(Y)
fosA7
country
Denmark
France
Germany
Netherlands
Poland
Spain

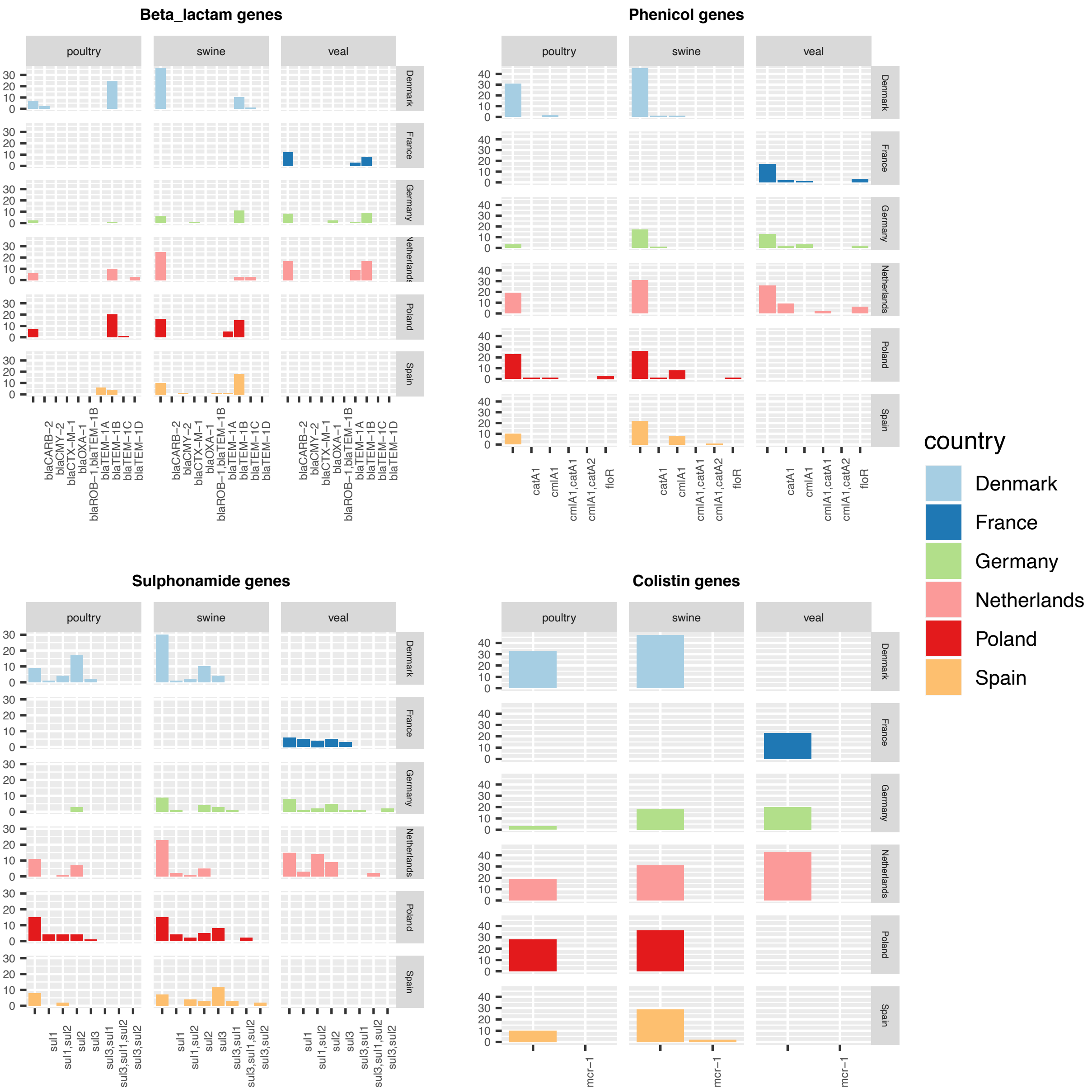
Beta_lactam genes
Phenicol genes
Sulphonamide genes
Colistin genes
poultry
swine
veal
Denmark
France
Germany
Netherlands
Poland
Spain
blaCARB−2
blaCMY−2
blaCTX−M−1
blaOXA−1
blaROB−1,blaTEM−1B
blaTEM−1A
blaTEM−1B
blaTEM−1C
blaTEM−1D
catA1
cmlA1
cmlA1,catA1
cmlA1,catA2
floR
sul1
sul1,sul2
sul2
sul3
sul3,sul1
sul3,sul1,sul2
sul3,sul2
mcr−1
country
Denmark
France
Germany
Netherlands
Poland
Spain

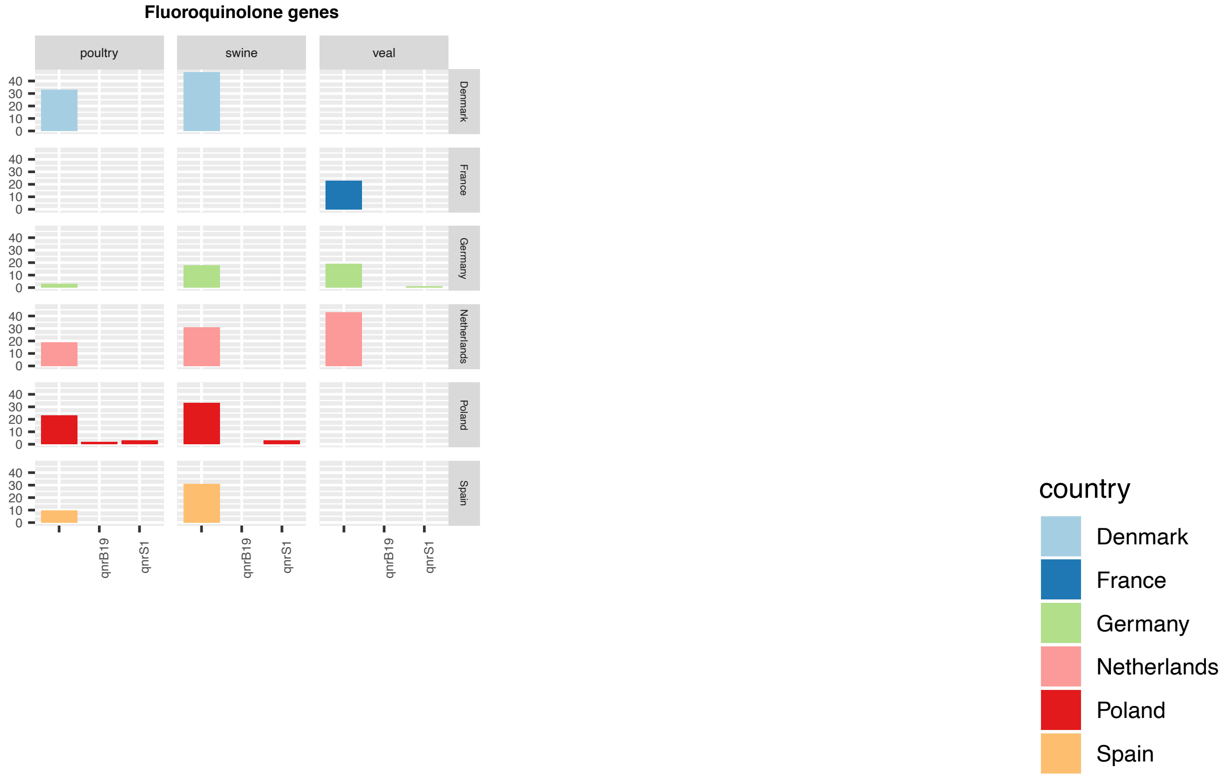

Supplementary figure 12: Distribution of resistance and susceptible *E. coli* isolates in different AMR genes and host. Y-axis is number of isolates. Three columns are number of isolates from poultry, swine and veal. In each column, first bar legend is number of isolates that do not carry AMR genes. Due to large number of genes, the distribution of AMR genes from aminoglycoside cannot be shown in this figure.

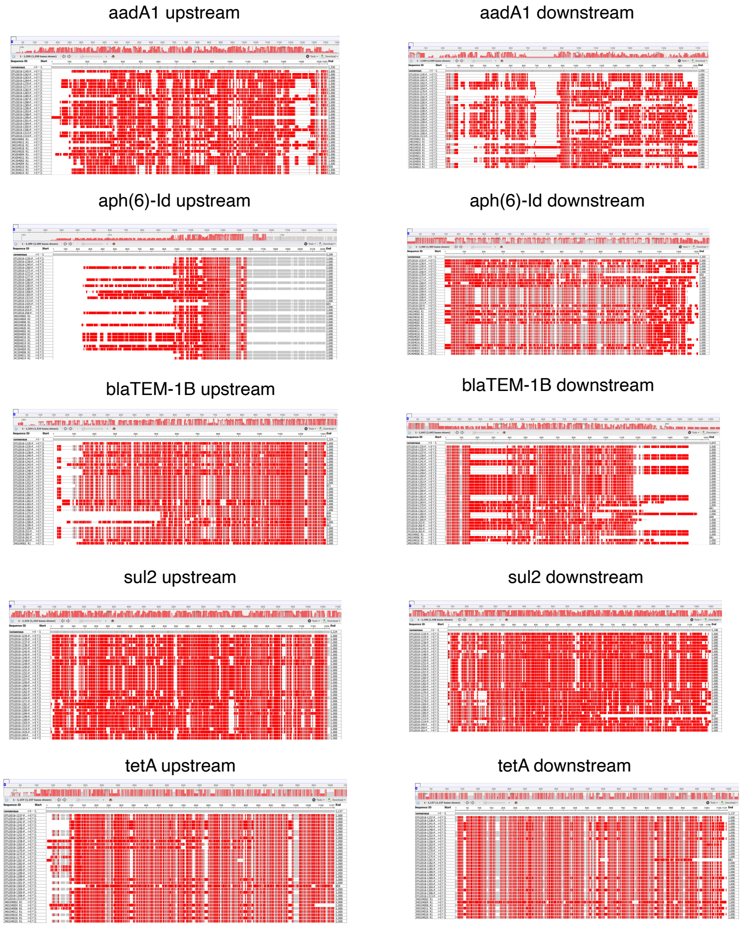

Supplementary figure 13: Multiple alignment of 1 kb upstream and 1 kb downstream of the most abundance 5 AMR genes using MUSCLE alignment and NCBI multiple sequence alignment viewer 1.14.1 for visualization. Due to visual practicality, the alignment showed only partial set of *E. coli* genomes. Grey colour represents conserved region in all 627 *E. coli* genomes. Red colour represents variable region.

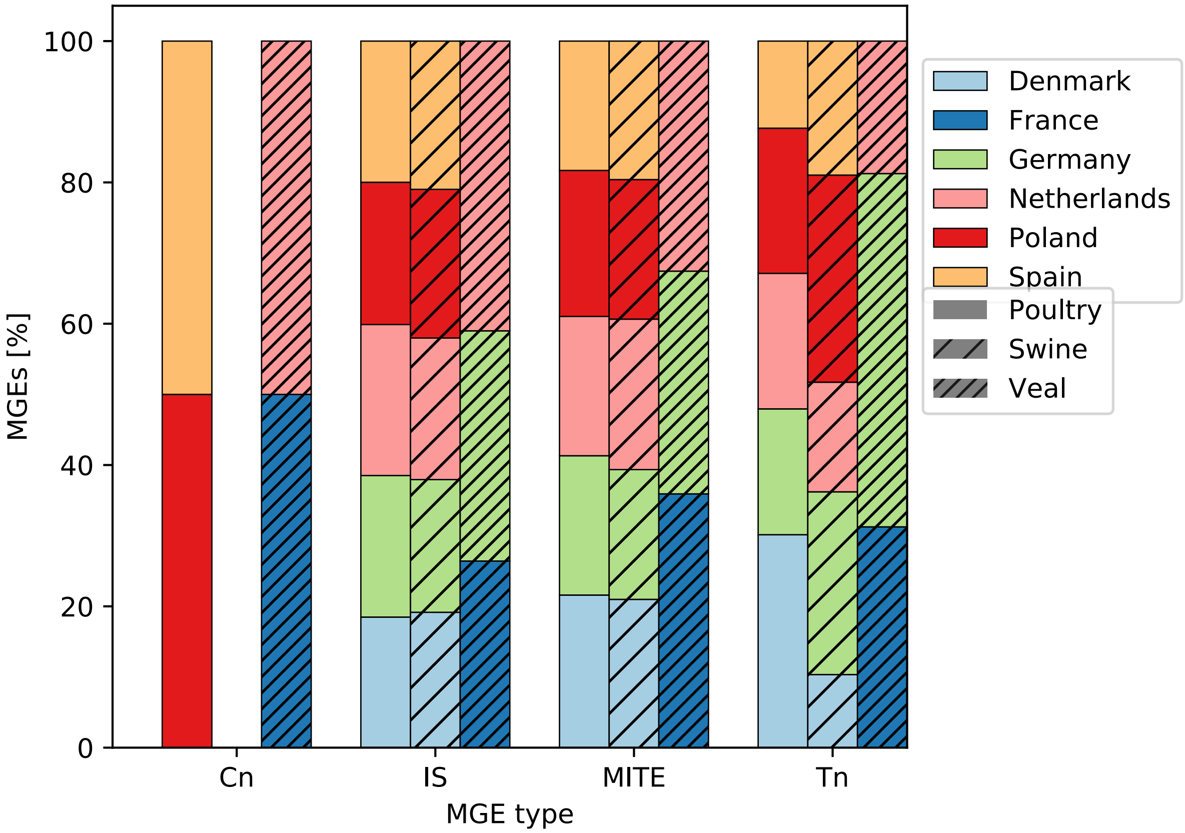

Supplementary figure 14: Composition of MGEs per country and host.

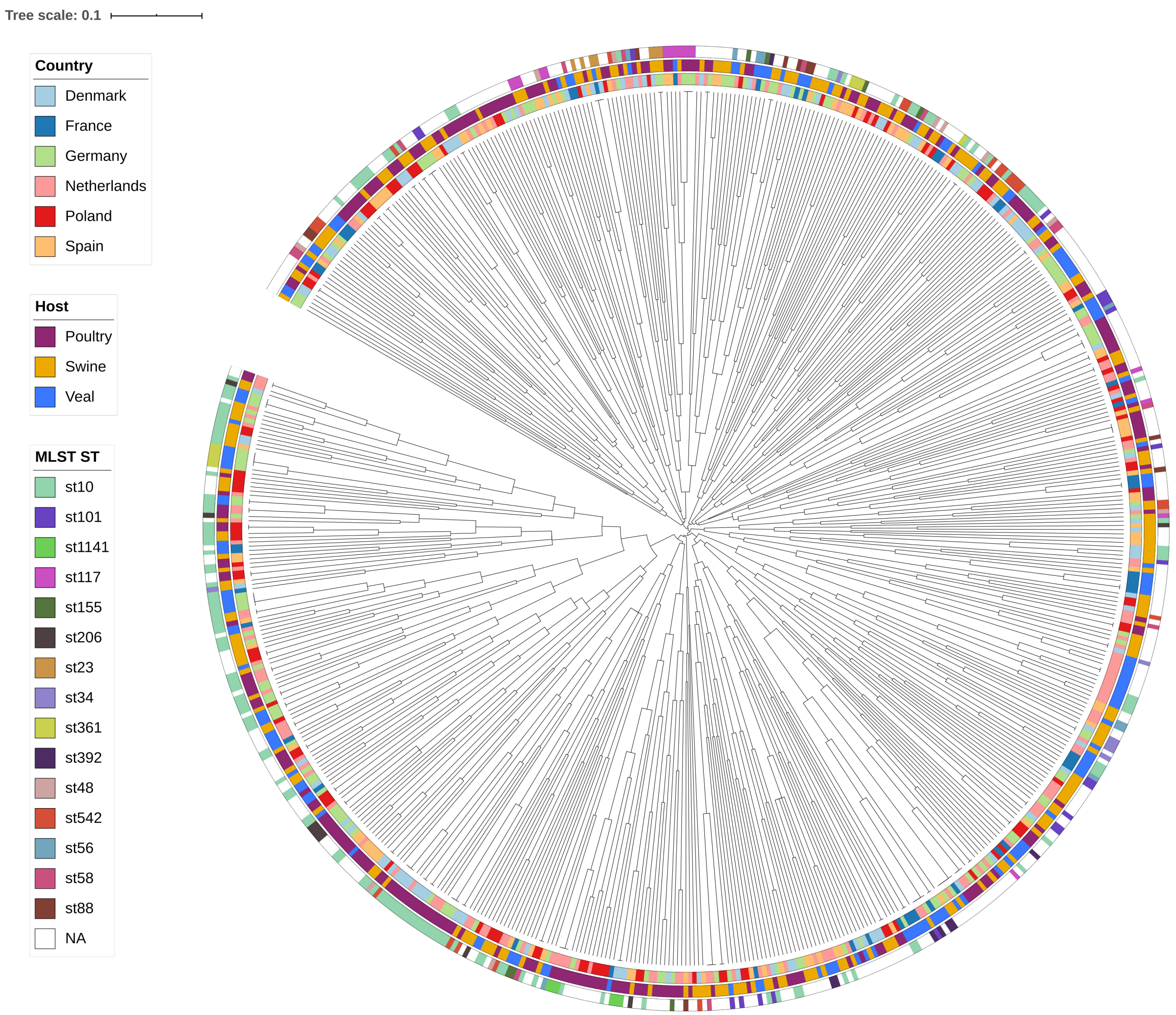

Supplementary figure 15: Tree based on MGE profiles from all *E. coli* isolates. The legends from inner circle to outer circle are country, host, and ST type. The 15 most common ST type were displayed.

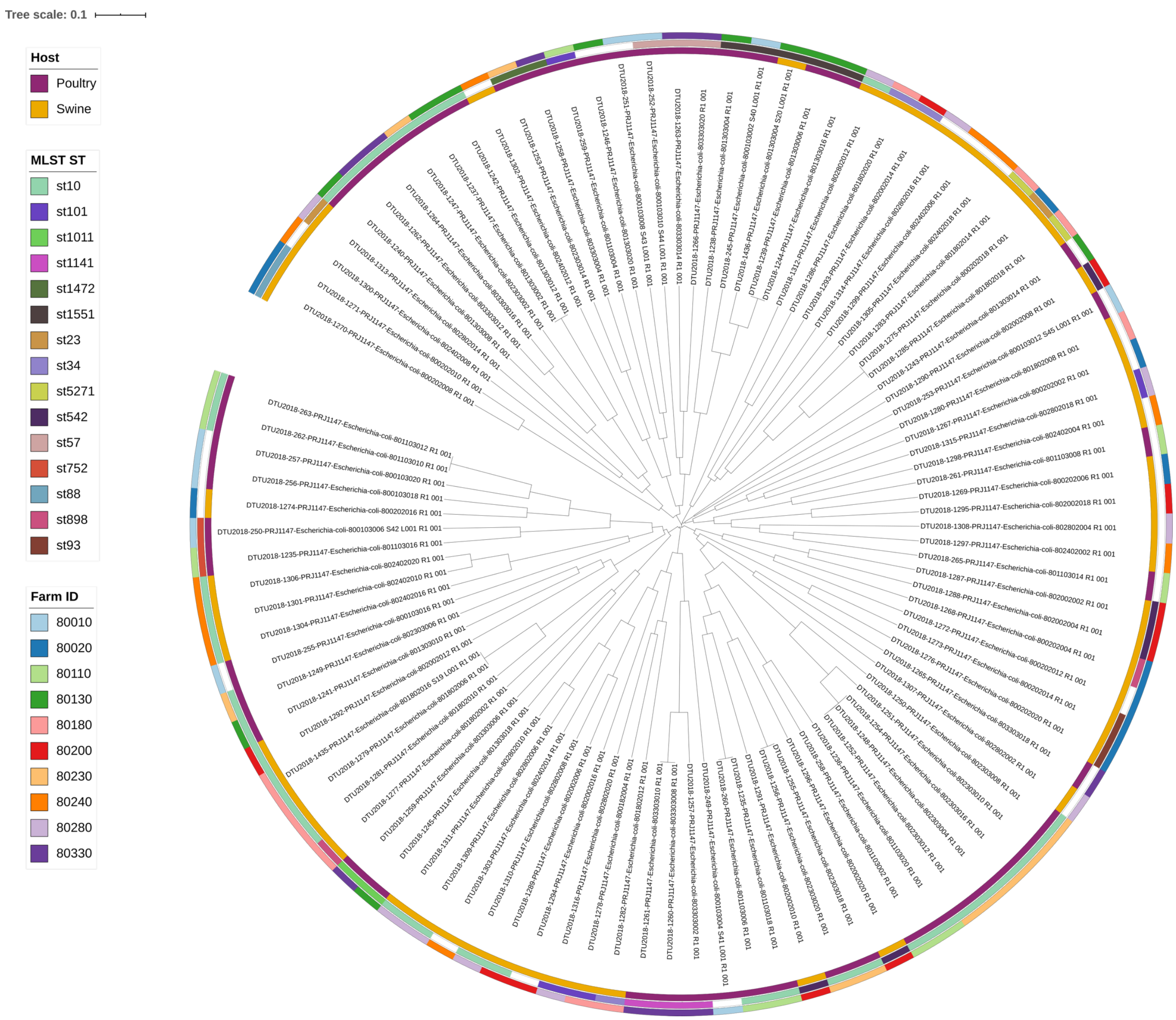

Supplementary figure 16: Tree based on MGE profiles of *E. coli* isolates from Denmark. The legends from inner circle to outer circle are host, ST type and farm ID.

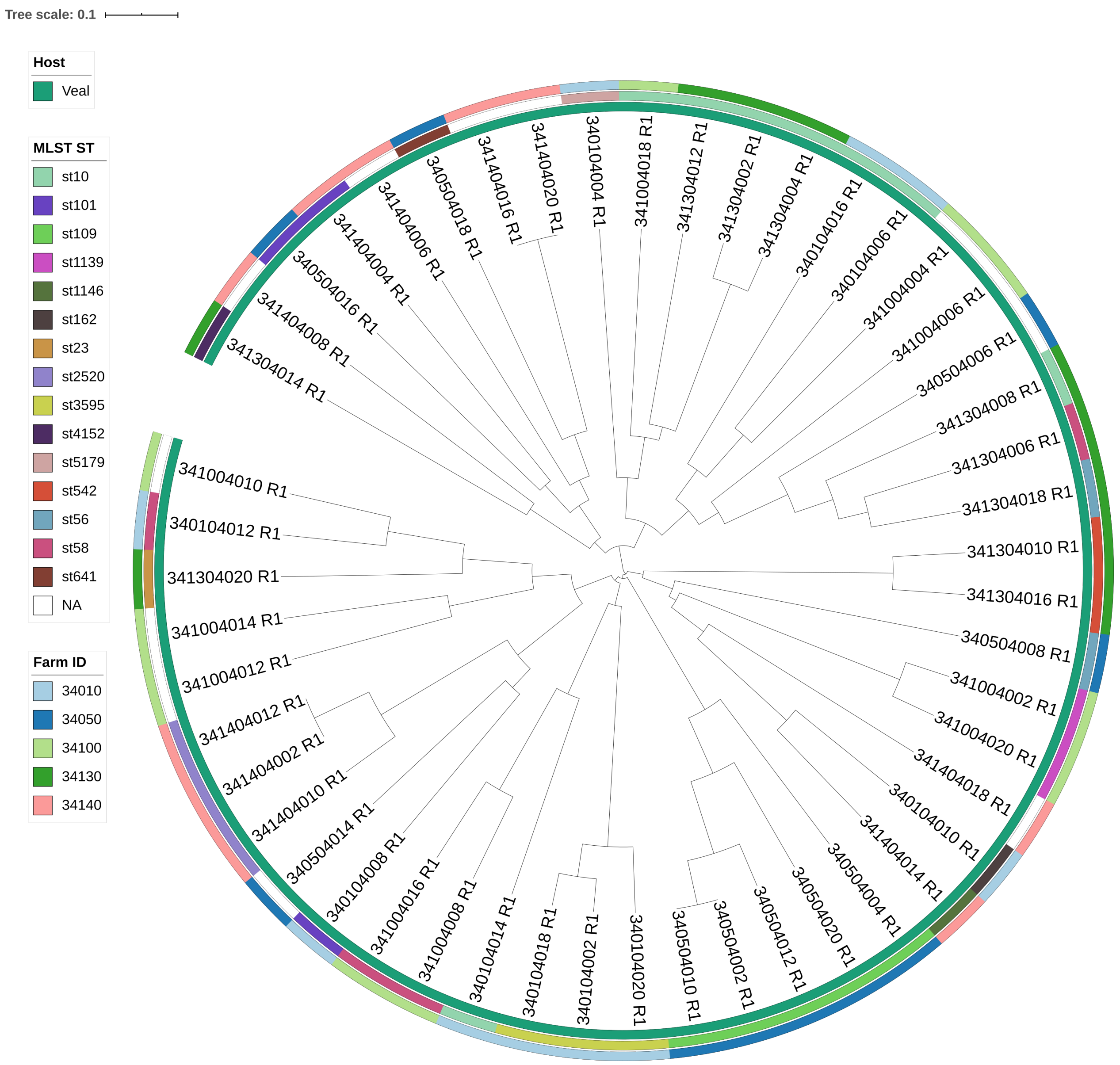

Supplementary figure 17: Tree based on MGE profiles of *E. coli* isolates from France. The legends from inner circle to outer circle are host, ST type and farm ID.

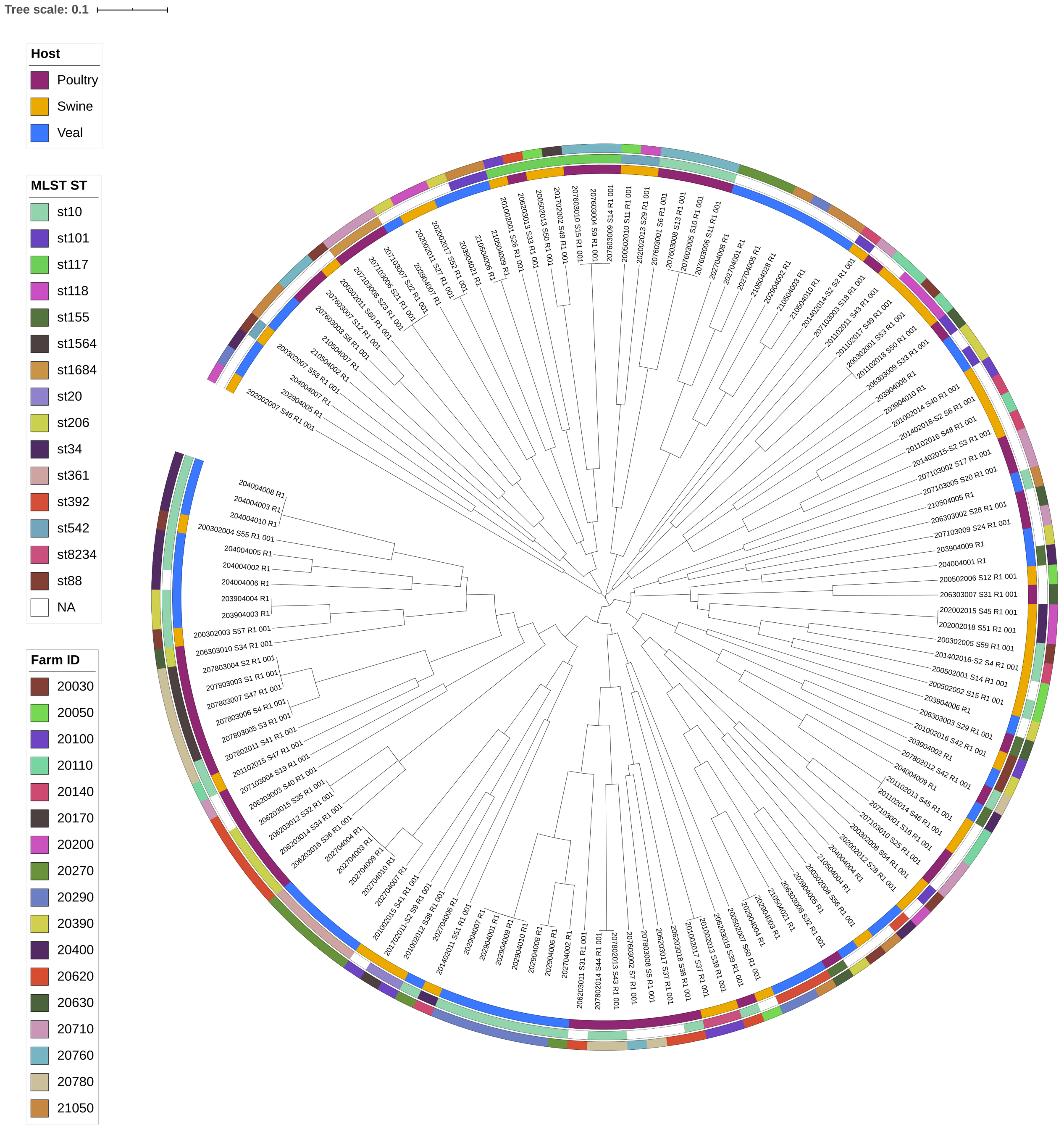

Supplementary figure 18: Tree based on MGE profiles of *E. coli* isolates from Germany. The legends from inner circle to outer circle are host, ST type and farm ID.

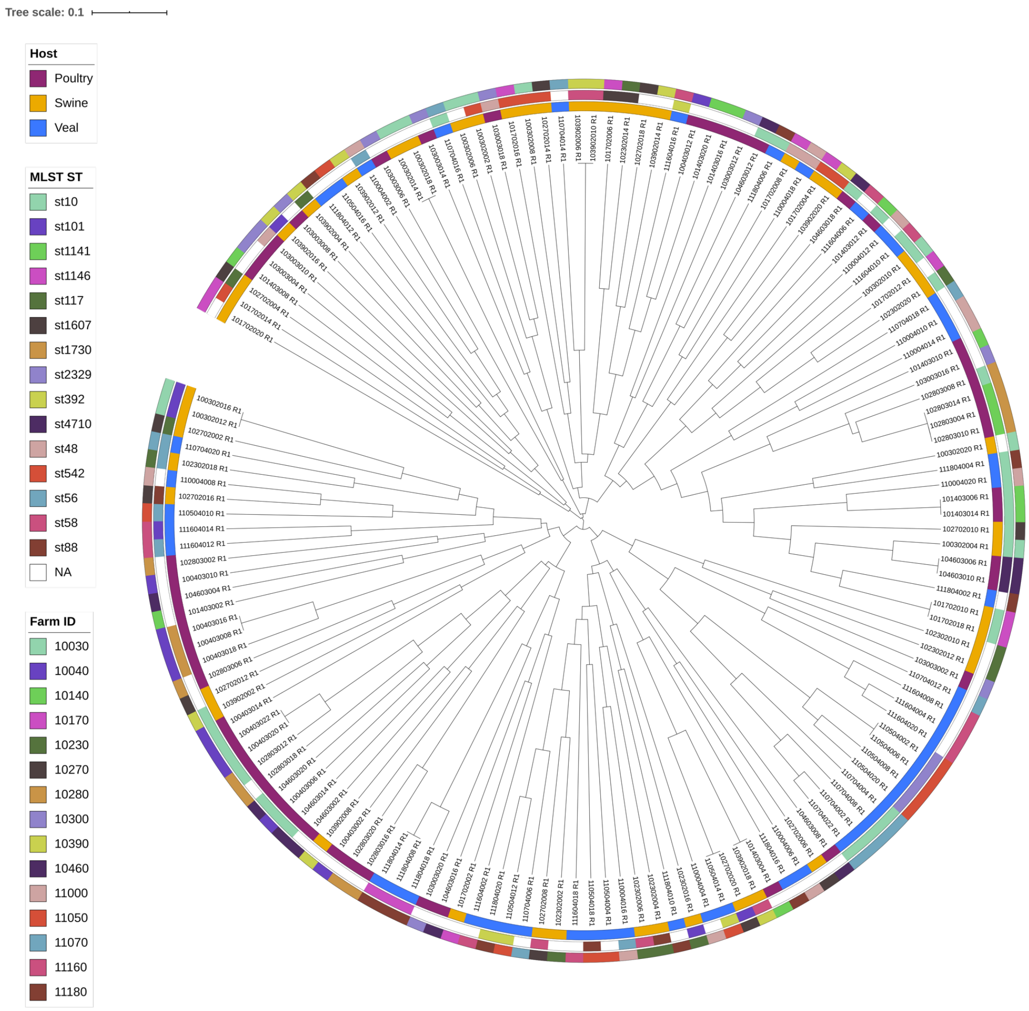

Supplementary figure 19: Tree based on MGE profiles of *E. coli* isolates from The Netherlands. The legends from inner circle to outer circle are host, ST type and farm ID.

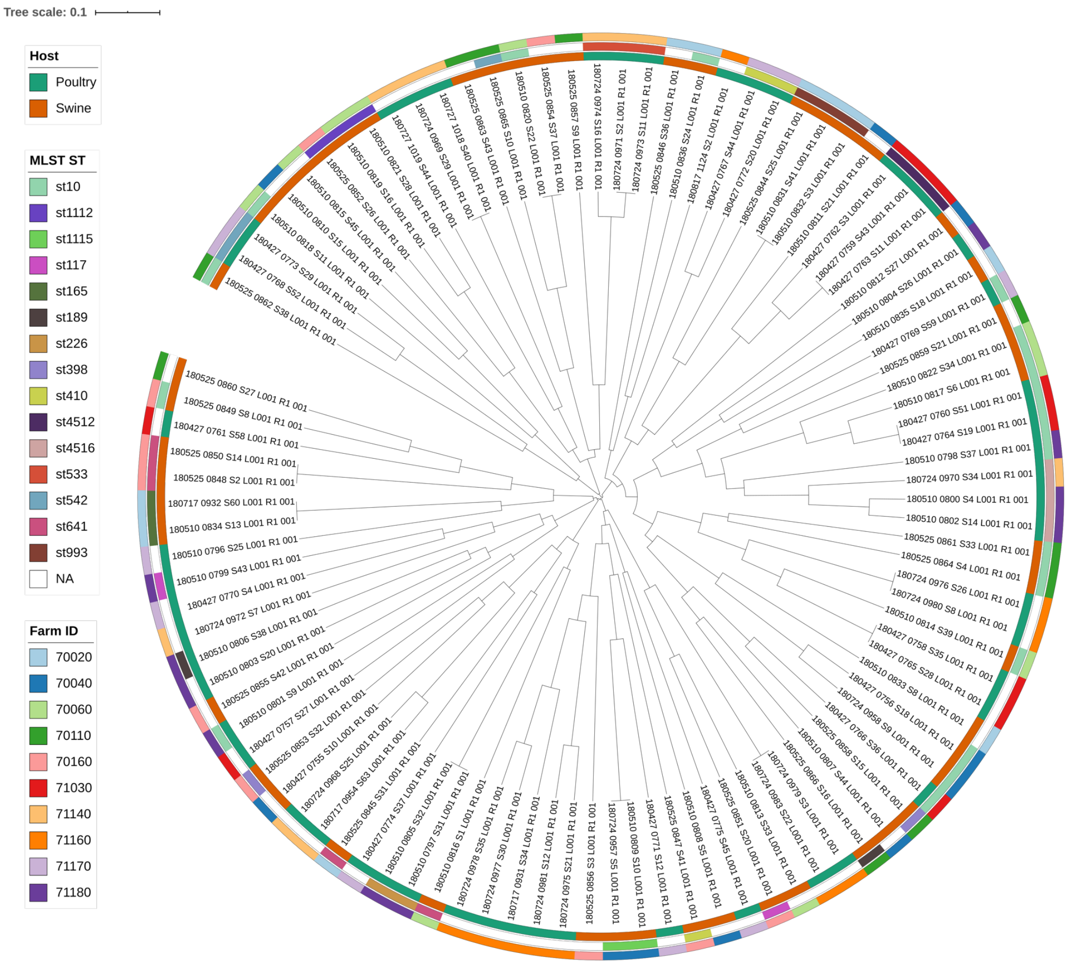

Supplementary figure 20: Tree based on MGE profiles of *E. coli* isolates from Poland. The legends from inner circle to outer circle are host, ST type and farm ID.

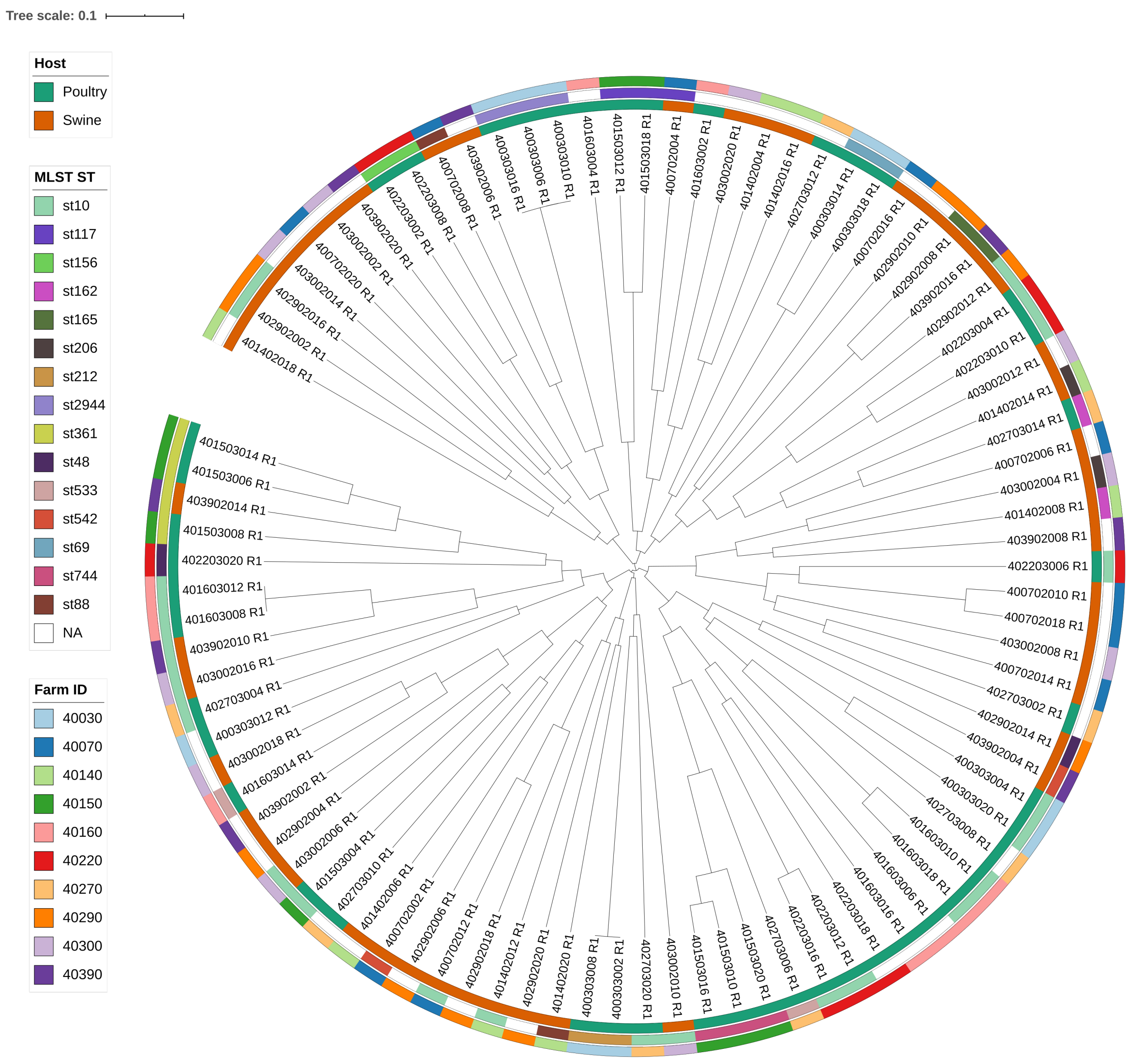

Supplementary figure 21: Tree based on MGE profiles of *E. coli* isolates from Spain. The legends from inner circle to outer circle are host, ST type and farm ID.

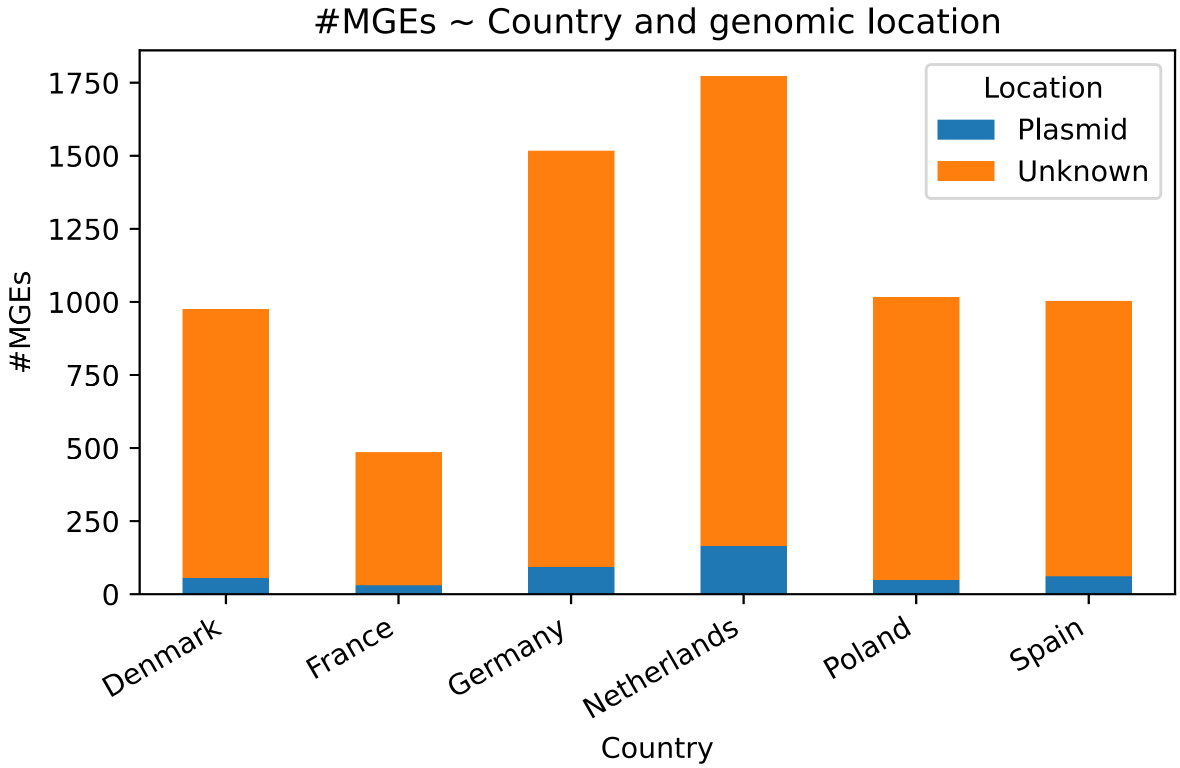

Supplementary figure 22: Distribution of MGEs carried on plasmids by country.

Supplementary Table 1: List of strains, metadata, genomic information and ENA accession number (excel file).

Supplementary table 2: Ten most frequent insertion sequences including number of observations, the percentage of isolates they were observed in and average copy number of complete and truncated MGEs.

**MGE name**	**MGE family**	**#MGEs**	**MGE frequency (%)**	**Avg copy number of complete IS**	**Avg copy number of truncated IS**
ISEc1	ISAs1	591	87.9	1.3	3.4
IS609	IS200/IS605	537	79.9	1.3	1.9
ISEc30	IS1	319	47.5	1.1	1.7
ISKpn8	IS3	311	46.3	1.0	1.1
IS26	IS6	246	36.6	1.1	4.5
ISEc38	ISL3	237	35.3	1.0	1.3
IS629	IS3	228	33.9	1.1	2.2
IS30	IS30	208	31.0	1.0	3.2
IS3	IS3	182	27.1	1.0	3.3
ISEc5	ISAs1	181	26.9	1.0	1.3

Supplementary table 3: The ten insertion sequences with the highest average copy number variation of truncated elements. The table includes the MGE family and the average copy number of complete and truncated elements.

**MGE name**	**MGE family**	**Avg copy number of complete IS**	**Avg copy number of truncated IS**
ISKpn14	IS1	1.0	32.6
ISApl1	IS30	1.0	13.8
ISKpn26	IS5	1.0	7.6
ISAs22	IS3	1.0	6.0
ISPan1	IS5	0.0	5.9
IS5	IS5	1.0	5.7
IS903	IS5	1.1	4..8
IS26	IS6	1.1	4.5
ISKpn20	IS3	0.0	4.0
ISPeat2	IS3	1.0	3.5

Supplementary table 4: Resistance genes carried on transposon type MGEs.

MGE name	#MGEs	MGE type	Resistance gene
Tn2	111	unit transposon	blaTEM-1B, blaTEM-1C, blaTEM-52C, blaTEM-52B, blaTEM-55
Tn6082	20	unit transposon	-
Tn1000	4	unit transposon	-
Tn6196	2	unit transposon	-
Tn4352	1	composite transposon	Aph(3')-Ia

Supplementary table 5: Number of putative composite transposons predicted, the number that carried AMR genes, flanking MGEs, their length (including the flanking MGEs) and potentially carried AMR genes.

Name	#MGEs	#Carry AMR	Flanking IS	Total length	Resistance genes
cn_18514_ISVsa3	8	8	ISVsa3	18514	aadA1, blaOXA-1, floR, sul1
cn_15885_ISAba1	4	4	ISAba1	15885	aph(6)-Id, floR, sul2, tet(Y)
cn_19464_ISVsa3	4	4	ISVsa3	19464	aadA1, floR, sul1, dfrA1
cn_19497_ISVsa3	4	4	ISVsa3	19497	aadA1, ant(2'')-Ia, floR, sul1
cn_4420_IS26	2	2	IS26	4420	aadA2b, lnu(F)
cn_5331_IS26	2	2	IS26	5331	blaCTX-M-1, mph(A)
cn_2819_IS26	1	1	IS26	2819	dfrA8
cn_2824_IS26	1	1	IS26	2824	blaTEM-1B
cn_3684_IS26	1	1	IS26	3684	dfrA14
cn_5556_IS26	1	1	IS26	5556	blaTEM-1B
cn_7944_ISVsa5	1	1	ISVsa5	7944	tet(B)
cn_8224_ISVsa3	1	1	ISVsa3	8224	floR
cn_8743_ISVsa5	1	1	ISVsa5	8743	tet(B)

Supplementary table 6: Variability of MGE associated arrays of AMR genes.

Arrays	N combinations	MGE name	Distance
aph(6)-Id	9	IS1133	873
aph(6)-Id, tet(A)	2	IS1133	873
aph(6)-Id, tet(A), dfrA1	1	IS1133	873
sul3	3	IS26	2022
sul3, aadA1, cmlA1, aadA2, dfrA12	2	IS26	2022
sul3, aadA1, cmlA1, aadA2b	1	IS26	2022
sul3, aadA1, dfrA1	2	IS26	2022
sul3, aadA2, dfrA12	1	IS26	2022
mph(A)	7	IS6100	2259
mph(A), blaTEM-1B, qnrS1	1	IS6100	2259
aac(3)-VIa	1	ISEc58	4151
aac(3)-VIa, aadA1	4	ISEc58	4151
aph(4)-Ia	5	ISEc59	368
aph(4)-Ia, aac(3)-IV	1	ISEc59	368
aph(4)-Ia, aac(3)-IV, sul3	1	ISEc59	368
aph(4)-Ia, aac(3)-IV, sul3, aadA1, cmlA1, aadA2b	1	ISEc59	368
sul2	13	ISVsa3	553
sul2, blaTEM-1B	16	ISVsa3	553
